# *In situ* monitoring of ligand-to-metal energy transfer in combination with synchrotron-based X-ray diffraction methods to elucidate the synthesis mechanism and structural evolution of lanthanide complexes

**DOI:** 10.3389/fchem.2025.1536383

**Published:** 2025-04-17

**Authors:** Ban H. Al-Tayyem, Philipp Müscher-Polzin, Kanupriya Pande, Oleksandr Yefanov, Valerio Mariani, Anja Burkhardt, Henry N. Chapman, Christian Näther, Michael Braun, Marvin Radke, Steve Waitschat, Kenneth R. Beyerlein, Huayna Terraschke

**Affiliations:** ^1^ Institut für Anorganische Chemie, Christian-Albrechts-Universität zu Kiel, Kiel, Germany; ^2^ Center for Free-Electron Laser Science CFEL, Deutsches Elektronen-Synchrotron DESY, Hamburg, Germany; ^3^ Deutsches Elektronen-Synchrotron DESY, Hamburg, Germany; ^4^ Centre for Ultrafast Imaging, Hamburg, Germany; ^5^ Department of Physics, University of Hamburg, Hamburg, Germany

**Keywords:** *In situ* luminescence, ligand-to-metal energy transfer, lanthanide complexes, crystal structure, small-molecule serial crystallography, synchrotron radiation

## Abstract

Despite wide application of lanthanide complexes in solar cells, light-emitting diodes and sensors, their crystallization mechanisms have not been studied in detail. Further investigations of this kind can lead to the development of targeted synthesis protocols and tailoring of their structure-related physical properties. In this work, the structural evolution during the synthesis of the luminescent [Tb (bipy)_2_(NO_3_)_3_] (bipy = 2,2′-bipyridine) complex is studied by monitoring the ligand-to-metal energy transfer through *in situ* luminescence measurements combined with synchrotron-based X-ray diffraction (XRD) analysis. These experiments reveal an interesting crystallization pathway involving the formation of a reaction intermediate that is dependent on parameters such as ligand-to-metal molar ratios. In addition, the structure of [Tb (bipy)_2_(NO_3_)_3_] is solved from serial crystallography data collected at a microfocused synchrotron X-ray beamline. This is an emerging technique that can be used to interrogate individual crystallites and overcome beam damage effects. The resulting structure is found to correspond to that determined by classical single crystal XRD, and a perspective on realizing future *in situ* measurements of this type is given. This work therefore describes multiple advancements combining crystallite-specific diffraction probes and *in situ* techniques to track the synthesis kinetics of luminescent materials.

## 1 Introduction

The worlds growing energy and technological demands requires the synthesis of new functional materials (Kuznetsov and Edwards, 2010). While it is well known that material properties are strongly dependent of their crystal structure, structure determination and control of the crystallization process can be a bottleneck in materials development. Despite the many advantages of single-crystal and *in situ* powder X-ray diffraction measurements, these techniques have required samples to be highly crystalline to follow the crystallization processes, with a large crystal size for structure determination. We will present two techniques to complement these approaches, namely, serial crystallography (Nam, 2020) for solving inorganic small molecule crystal structures and *in situ* measurements for monitoring the crystallization pathway (Terraschke et al., 2018; Lindenberg et al., 2019).

Serial X-ray crystallography involves the use of a bright, tightly focused X-ray source, collecting a large set of short-exposure diffraction patterns and using the intensities merged from thousands of snapshot diffraction images from different crystallites to solve the structure (Chapman et al., 2011; Boutet et al., 2012). Such macromolecular structure solution has been shown to be possible from small crystals (Gati et al., 2017), and by *de novo* phasing (Barends et al., 2014; Yamashita et al., 2015; Colletier et al., 2016). Furthermore, it has been used to investigate the light-induced dynamics of photoactive proteins (Tenboer et al., 2014; Barends et al., 2015; Nango et al., 2016) and diffusion-limited reactions of macromolecules with ligands (Stagno et al., 2017; Kupitz et al., 2017; Beyerlein et al., 2017a). This approach has been recently extended to small-molecule serial femtosecond X-ray crystallography (smSFX), which has allowed for the structure solution of radiation sensitive inorganic complexes (Schriber et al., 2022; Aleksich et al., 2023) and study of their light-induced atomic dynamics (Kang et al., 2024; Støckler et al., 2023).

Monitoring the formation of solid materials *in situ* is important for unraveling the phenomena behind the crystallization process (Derelli et al., 2024; Embrechts et al., 2018; Pienack and Bensch, 2011; Keilholz et al., 2023). Important examples of such phenomena are the desolvation of ions in solution and their incorporation by the formed nuclei as well as formation of reaction intermediates during phase transitions. This knowledge combined with the influence of synthesis parameters such as reagent concentrations or reaction temperature on the phenomena listed above are essential for controlling and improving structure-related properties of functional materials (Pienack et al., 2016; Arana et al., 2017; Rönfeldt et al., 2020; Pienack et al., 2018). Important examples of functional materials are luminescent complexes (Suckert et al., 2017; Neumann et al., 2017; Cotton et al., 2003; Cotton and Raithby, 2017), crucially important for technological and biomedical applications such as production of light-emitting diodes (LEDs) (Li et al., 2020) fluorescent chemosensor (Cable et al., 2013; Binnemans, 2009; Zhou et al., 2020), medical diagnostic (Heffern et al., 2014; Bünzli, 2010; Dong et al., 2015; Wen et al., 2019) and treatment (Marturano et al., 2019). They have been widely studied due to their unique photoluminescent properties, i.e., sharp emission lines, long lifetime and the ability to emit light in the visible region (Binnemans, 2009). For example, the luminescent complex [Tb (bipy)_2_(NO_3_)_3_] was first synthesized in 1969 and its unit cell was studied by single crystal X-ray diffraction collected by the Weissenberg method. [Tb (bipy)_2_(NO_3_)_3_] was found to crystalize in *Pbcn* orthorhombic space group with Z = 4. In this complex, Tb^3+^ ions have 10-fold coordination with four nitrogen from the two bidentate bipyridine ligands and with three bidentate nitrate groups (Moss and Sinha, 1969).

We report on the synthesis, spectroscopy and structure of the compound [Tb (bipy)_2_(NO_3_)_3_]. Here, we have solved the structure of the compound as a model system from a serial crystallography measurement of an ensemble of microcrystals. Our [Tb (bipy)_2_(NO_3_)_3_] structure is found to agree with that found from new single crystal X-ray diffraction analysis of crystals of approximately 1 mm. This affirmation demonstrates the large data volume needed for *ab initio* direct methods structure solution of inorganic compounds. Presented data on the obtained unit cell distribution in the ensemble also suggests how information about the homogeneity and purity of a powder sample can be obtained from such a measurement. In addition, the synthesis of this compound is studied using *in situ* fluorescence spectroscopy and synchrotron-based powder X-ray diffraction. We then conclude the article with a perspective of combining serial and fluorescence measurements to track the synthesis of such compounds *in situ*. Such a crystallite-sensitive tracking of a reaction could offer new insights into materials synthesis and how to govern their crystallization processes.

## 2 Experimental section

### 2.1 Materials

2,2ʹ-Bipyridine (99+%, Alfa Aesar GmbH and Co. KG, Karlsruhe, Germany) and Tb(NO_3_)_3_·5H_2_O (99.99%, Abcr, Karlsruhe, Germany) were of analytical grades and used as received without further purification.

### 2.2 Sample preparation

#### 2.2.1 Synthesis for *ex situ* characterization

[Tb (bipy)_2_(NO_3_)_3_] was firstly synthesized *ex situ* (Table 1, Experiment 1) by adding 4 mL of the 2,2ʹ-bipyridine solution (100 mM) to a 4 mL of Tb(NO_3_)_3_·5H_2_O solution (50 mM). The resultant solution was left to react at room temperature until the product precipitated as colorless crystals (0.1038 g, yield 79%).

**TABLE 1:** Experimental conditions for elucidating the formation mechanism of the [Tb (bipy)_2_(NO_3_)_3_] complex, *in situ* applying setups A (University of Kiel) or B (DESY P08 beamline) as well as *ex situ*.

Exp. Number	Tb^3+^ concentration/M	Tb^3+^ volume/mL	bipy concentration/M	bipy volume /mL	$\dot{V}$ (bipy)/mL⋅min^−1^	bipy:Tb^3+^ molar ratio	λ_ex_ /nm	Setup
1	0.05	4	0.10	4	—	2:1	365	*ex situ*
2	0.02	30	0.28	5	0.5	2:1	365	A
3	0.02	30	0.28	5	10	2:1	365	A
4	0.07	40	1.12	5	0.5	2:1	365	B
5	0.07	40	0.84	5	0.5	1.5:1	365	B
6	0.07	40	0.56	5	0.5	1:1	365	B

#### 2.2.2 Synthesis for *in situ* characterization

[Tb (bipy)_2_(NO_3_)_3_] was synthesized using a simplified co-precipitation method as described in our reported studies (Polzin et al., 2018). The analytical study of the complex formation was performed *in situ* using different monitoring techniques under different synthetic parameters (Table 1, Experiments 2-6). All experiments were executed in a glass reactor at room temperature, in which the metal precursor was dissolved in ethanol and stirred at 500 rpm. The ethanolic ligand solution was then controllably added to the metal solution at rates of 0.5 or 10 mL min^−1^. At predetermined times, samples were removed from reaction vessel for further *ex situ* analysis, the samples collected were directly quenched, centrifuged and dried at 80°C for 2 h. The *in situ* study was carried out using two different setups as we previously reported (Ströh et al., 2023; Ströh et al., 2024). Experiments using Setup A (Table 1, Experiment 2-3) were carried out at Kiel University while experiments using Setup B (Supplementary Table S1, Experiment 4-6) were conducted at the P08 beamline of the Deutsches Elektronen-Synchrotron (DESY).

Setup A combines *in situ* measurement of pH value, ionic conductivity and *in situ* luminescence. In this setup, the simultaneous *in situ* measurements were conducted using an EasyMax^®^ 102 (Mettler Toledo, Gießen, Germany) synthesis workstation. This workstation granted automatic control over the reaction parameters, e.g. the solution addition rate, reaction temperature and stirring speed. Additionally, it allowed simultaneous multiparameter monitoring of the solution pH value and ionic conductivity. The *in situ* luminescence measurements were recorded using portable EPP 2000 (StellarNet Inc., United States) spectrometer equipped with a charge-coupled device (CCD)-based detector and a FL322 Fluorolog-3 spectrofluorometer (HORIBA Jovin Yvon GmbH, Unterhaching, Germany), which contains an R928P Photomultiplier, iHR-320-FA triple grating imaging spectrograph and a 450 W xenon lamp. The detectors were attached to an optical fiber, which was submerged into the reactor solution while the reactor was irradiated by UV light-emitting diodes (LEDs) with a wavelength of 365 nm (Sahlmann Photochemical Solutions, Germany).

In Setup B (see Supplementary Figures S1, S2, supporting information), the *in situ* XRD and *in situ* luminescence measurements were simultaneously recorded. The XRD structural analysis was obtained using an *in situ* synchrotron-based crystallography technique. Radiation of 25 keV photon energy penetrated the glass reactor walls and the reaction solution, enabling these measurements. Additionally, the reactor was specially designed to have an indented tube in one of its walls to decrease the radiation pathway length through the reaction volume. This reactor was positioned in a holder containing two openings for the transmission of X-ray radiation, as well as a third large opening for the UV-light excitation source (Pienack et al., 2016). The setup contained an integrating stirring system and a ground plate that fits different beamlines without necessitating readjustment after each reaction. The X-ray outlet was equipped with an aluminum window, which Bragg peaks served as an external standard for normalizing the intensities of the XRD patterns and correcting for the intensity fluctuation of the X-ray beam. *In situ* XRD measurements were recorded in a transmission geometry every 30 s. At P08 (25 keV, λ = 0.04959 nm), a PerkinElmer XRD1621 detector (PerkinElmer Technologies, Walluf, Germany, 2048 × 2048 pixels, *x* pixel size of 200.00 mm, and *y* pixel size of 200.00 mm) was applied. In this setup, the *in situ* luminescence measurements were recorded using the same EPP2000 spectrometer and light sources applied in setup A. However, the reaction volume used in Setup B experiments was increased to 40 mL to reach the minimum solution height required for accurate *in situ* XRD measurements. The volumes and concentrations of the solutions used in this work are summarized in Table 1.

For the *ex situ* characterization, luminescence spectra were measured using a Fluorolog-3 with a R928P photomultiplier, whereas *ex situ* XRD patterns were recorded using a STOE Stadi-p powder diffractometer (Cu-Kα1 radiation, λ = 1.540598 Å) equipped with a Ge monochromator and a DECTRIS^®^ MYTHEN 1K detector (DECTRIS, Baden-Daettwil, Switzerland). Additionally, the reflection spectra were measured at room temperature with Varian Techtron PtyUV/Vis/NIR two-channel Cary 5,000 spectrometer using BaSO_4_ as a reference. The single-crystal XRD analysis was performed using an IPDS-2 diffractometer (STOE and Cie GmbH, Darmstadt, Germany) with Mo-Kα radiation (λ = 0.71073 Å).

### 2.3 Serial X-ray crystallography structure solution

A sample was crystallized to the final product according to the protocol described in Section 2.2.2. This resulted in a powder with an average agglomerate size of approximately 1 µm.

**FIGURE 1:**
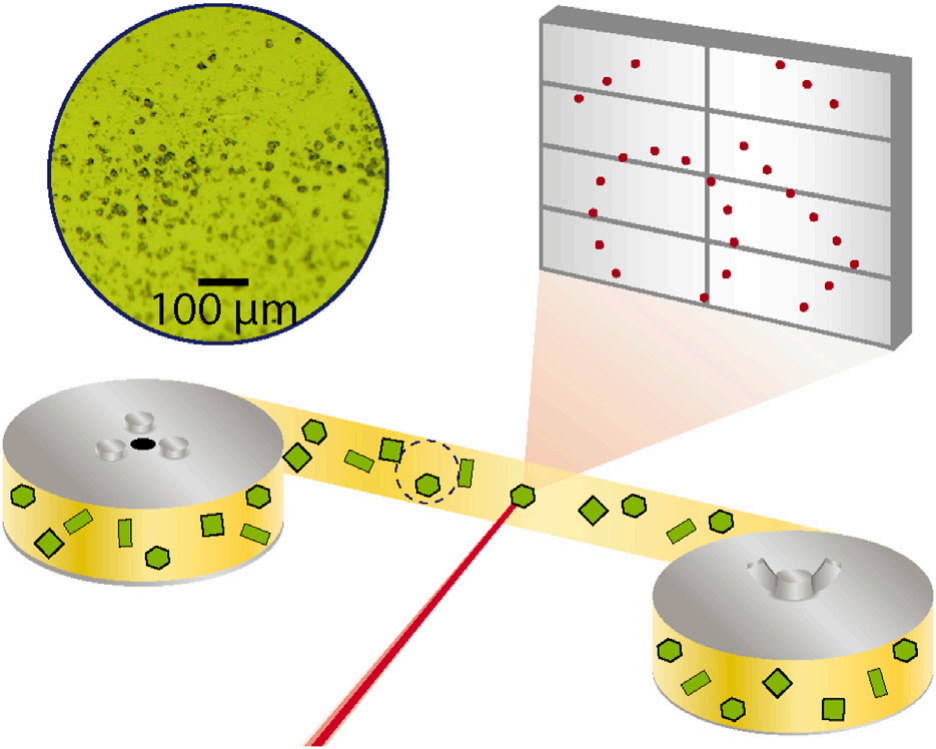
Serial crystallography measurement using polyimide tape drive. The red X-ray beam is focused onto a yellow polyimide tape that is strung between two rollers and contains green [Tb (bipy)_2_(NO_3_)_3_] crystals affixed to it. The diffraction from a crystal is measured in transmission on a large grey 2D detector. A microscopic image of such a tape that was used for the measurement is shown.

A serial crystallography dataset was measured at beamline P11 (20 keV, λ = 0.062 nm) of the PETRA III synchrotron (Burkhardt et al., 2016). The dry sample was fixed between 2 strips of polyimide tape that were each 6 mm wide and 12 µm thick (Caplinq, Netherlands). A uniform and dilute coverage of the sample powder was achieved by dusting it onto the sticky side of the tape and pulling off excess powder with another piece of tape. This process was followed to prepare a 2-m length of tape with [Tb (bipy)_2_(NO_3_)_3_] that was then affixed to a roll of non-sticky polyimide tape. This was mounted onto a tape drive device installed at the beamline that has been also used to conduct serial crystallography measurements of proteins (Beyerlein et al., 2017a). A schematic of the measurement geometry is shown in Figure 1.

The measurement was conducted with the sample at room temperature and using a 20 keV X-ray beam focused to 4 × 8 µm with a flux of 10^13^ photons/second. The sample tape was then fed through the tape drive device translating crystals through the X-ray focus at a constant rate of 0.7 mm/s. A spinning metal disc with holes in it served as an X-ray beam chopper and was installed upstream from the X-ray focusing optics and provided an exposure time of 9.3 ms at a repetition rate of 10 Hz. A Pilatus 6M detector was synchronized with the X-ray exposure and the resulting detector frames were saved for later evaluation. A live analysis of the fraction of recorded images found to contain a diffraction pattern (hit fraction) was made using the OnDA software (Mariani et al., 2016). A hit fraction around 50% was controlled during the experiment by changing the sample coverage density on the tape. When the end of the prepared sample tape was reached, the tape was rewound, translated a few beam diameters away from the previous height and fed through again. This rastering was repeated until the full width of the sample tape was scanned, after which another sample was prepared.

After the dataset was collected, the raw data images were again analyzed to sort out the diffraction images from blanks using a program called OffDA. Then the refined hit images were indexed using the *indexamajig* program of CrystFEL (White et al., 2012; White et al., 2013; White, 2014; White et al., 2016). Initially, this was used to index the patterns without providing unit cell information to the auto-indexing program DIRAX (Duisenberg, 1992). A dominant lattice consisting of a primitive orthorhombic cell with constants of a = 16.71 Å, b = 9.14 Å and c = 15.07 Å was found. This cell was then given as input to the FELIX indexer (Beyerlein et al., 2017b), which can independently index the patterns from multiple crystals in a snapshot image. The parameters of FELIX were then optimized to maximize the number of indexed crystals from the measured “hit” images. This resulted in a total of 288,396 indexed crystals in 186,432 frames, an average of 1.5 crystals per indexed frame. An overview of other dataset statistics is given in Table 2.

**TABLE 2:** Serial crystallography dataset statistics.

Collected images	891,102
Indexed images	186,432
Indexed crystals	288,396
Resolution range (Å)	16.70–0.7
Merging R_split_/CC*	2.46/0.999
Overall SNR	24.1
Completeness	100%
Refined R_1_/GOF	4.41/1.07

The integrated intensities from the indexed crystals were then merged using the CrystFEL program *process_hkl* assuming the *mmm* Laue class*.* The self-consistency of the merged intensities was quantified by randomly sorting the indexed crystals into two datasets of equal length and merging them independently. The difference between these datasets can then be quantified by calculating $R_{split}=2^{1/2}\sum \left(I_{1}-I_{2}\right)/\sum \left(I_{1}+I_{2}\right)$ , where the sum is carried out over all *hkl* reflections, and $I_{1}$ and $I_{2}$ are the intensities from each dataset. The trend of $R_{split}$ (Section 3.1) was used to determine the resolution cutoff of 0.7 Å (14 1/nm) for the merged intensities. The CrystFEL *hkl* file was then converted using the CCP4 programs *f2mtz* and *mtz2various* (Winn et al., 2011).

The merged intensities were analyzed by the program XPREP (Bruker) and the most likely space group was found to be *Pbcn*. The structure was then solved *ab initio* using shelxt (Sheldrick, 2015) and refined in the program olex2 (Dolomanov et al., 2009) using the olex2.refine program.

## 3 Results and discussion

### 3.1 [Tb (bipy)_2_(NO_3_)_3_] serial crystallography structure solution

**FIGURE 2:**
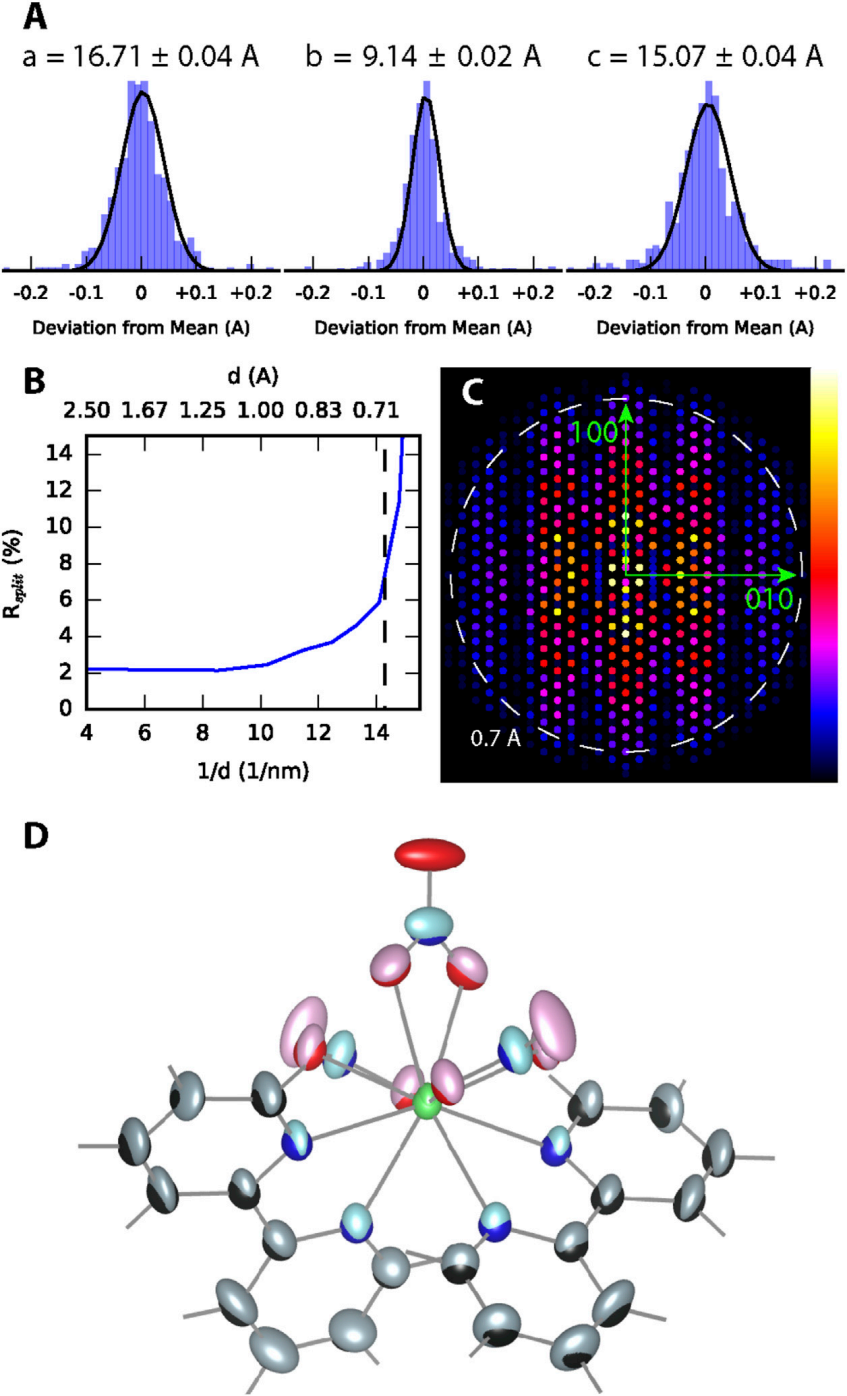
[Tb (bipy)_2_(NO_3_)_3_] Serial Structure Solution. **(A)** The distributions of orthorhombic unit cell parameters found by indexing frames without symmetry input are shown. **(B)** The trend of R_split_ for the merged intensities is shown along with a dashed line denoting the cutoff used on the reflection list. **(C)** A cross-section of the merged reflections through the h-k plane of reciprocal space, generated using the CrystFEL *render_hkl* program. Each spot represents an hkl reflection with the color representing the structure factor [sqrt(I)] according to the normalized color scale shown. **(D)** Structure comparison. The atomic anisotropic displacement isosurfaces obtained from serial crystallography (pastel colors) are overlayed onto those obtained from of the single crystal structure refinement (RGB colors).

The distribution of unit cell parameters determined from each indexed pattern is shown in Figure 2A and is in good agreement with those determined from the single crystal structure described later. The trend of the data quality metric *R*_*split*_ in Figure 2B shows that the merged intensities in the final dataset converged for scattering vector magnitudes below 14 nm^−1^. This was then used as a cutoff to define the reflection dataset used in structure solution. The dataset had 100% completeness in this range and a view of the merged intensities in the a*-b* reciprocal space plane is shown in Figure 2C. The structure of [Tb (bipy)_2_(NO_3_)_3_] determined *ab initio* from this dataset (pastel colors) is overlayed in Figure 2D with the single crystal structure described in detail in Section 3.2 (primary colors). From this figure it is seen that the atomic coordinates of the two structure solution methods are in good agreement, however, the anisotropic atomic displacement parameters of the serial crystallography structure are slightly larger. A higher sample temperature during the serial crystallography measurement (298 K) compared to the single crystal measurement (170 K) can partially explain this discrepancy. This could also be caused by merging intensity data from many crystals, which can combine different structural disorder found in each crystal.

As detailed in Table 2, the dataset used to solve the serial crystallography structure was formed from merging diffraction patterns from more than 288,000 crystallites. This required the collection of more than 890,000 patterns, which took more than 24 h of measurement time. When just 10,000 crystals from the serial dataset were merged and used to solve the structure, an incorrect space group of *Pna2*_*1*_ and a higher refined R-metric around 6% was found. Furthermore, the anisotropic thermal displacement parameters of the carbon atoms in the bipyridine ligands were double what was refined in the final structure. The temperature factors of other room temperature metal-bipyridine complex structures deposited in the Cambridge Structural Database were found to be in good agreement with those refined in the final structure.

This large amount of data was critical to obtaining an accurate structure as preliminary datasets prepared using fewer crystals resulted in structures with carbon and oxygen atoms missing in some places from ligands. Positional information about the Tb^3+^ center was possible with less data, however, the anisotropic displacement parameters of Tb^3+^ were found to be abnormally large. It should be noted that a higher number of crystals could be necessary for the solution of precursors or possible unknown intermediate phases with a decreased crystallite size and crystal quality, as in such cases the intensity of high-resolution Bragg reflections will be lower. This information is important for the design of future experiments to determine the nature of the early precursor phases suggested by our *in situ* optical measurements described below.

The necessity to merge data from almost 300,000 crystals presents a major challenge to performing *in situ* serial crystallography experiments to study these phases. Nonetheless, this result motivates efforts to develop instrumentation to increase the data collection speed and analysis routines to reduce the amount of data necessary to solve inorganic structures *ab initio* by serial crystallography. Faster collection is possible at modern XFEL facilities such as European XFEL and LCLS-II that have average repetition rates ranging from kHz to MHz. At this rate 890,000 patterns can be collected in a matter of seconds to minutes, allowing for a series of time points tracking the evolution of intermediate states to be collected in a few hours. However, our study also shows that the analysis of smSFX data is dramatically simplified when an X-ray photon energy above 15 keV is used. Compared to the sparse data collected from previous smSFX experiments (Schriber et al., 2022; Beyerlein et al., 2015), the resulting diffraction patterns contain enough Bragg spots and high-resolution information to use already developed auto-indexing and structure solution algorithms. The optimization of *in situ* serial crystallography experiments of this kind may require the high photon energies of 15–25 keV becoming available at superconducting-linac XFEL facilities. A new approach of convergent-beam serial crystallography provides the potential to access a reciprocal space volume of 50 times or more than with a collimated beam, in a single shot (Chapman et al., 2024). The patterns obtained using highly focused beams contain many more Bragg reflections, overcoming the problem of indexing sparse patterns and providing fully-integrated intensities. The approach is suitable for synchrotron and XFEL beamlines.

### 3.2 Solution of [Tb (bipy)_2_(NO_3_)_3_] crystal structure by single crystal X-ray diffraction

To confirm the serial crystallography results, [Tb (bipy)_2_(NO_3_)_3_] was synthesized *ex situ* (Table 1, Experiment 1), and its crystal structure of was solved by single crystal XRD analysis (Supplementary Tables S1–S3). As shown in Table 3, the unit cell parameters obtained by serial crystallography and single crystal XRD were similar and in agreement with previously reported results (Moss and Sinha, 1969). [Tb (bipy)_2_(NO_3_)_3_] crystallizes into the orthorhombic Pbcn space group (Supplementary Table S2) with a = 16.7014 (5) Å, b = 9.0291 (2) Å, c = 14.9826 (4) Å, and with four formula units in the unit cell (Z = 4).

**TABLE 3:** Cell parameters of [Tb (bipy)_2_(NO_3_)_3_] obtained by conventional single crystal XRD and serial crystallography in comparison.

Cell parameter	Single crystal XRD (at 170 K)	Serial crystallography (at 298K)
a [Å]	16.7014 (5)	16.704 (3)
b [Å]	9.0291 (2)	9.0291 (18)
c [Å]	14.9826 (4)	14.983 (3)
α, β, γ [°]	90	90
V [Å³]	2259.36 (10)	2259.7 (8)

**FIGURE 3:**
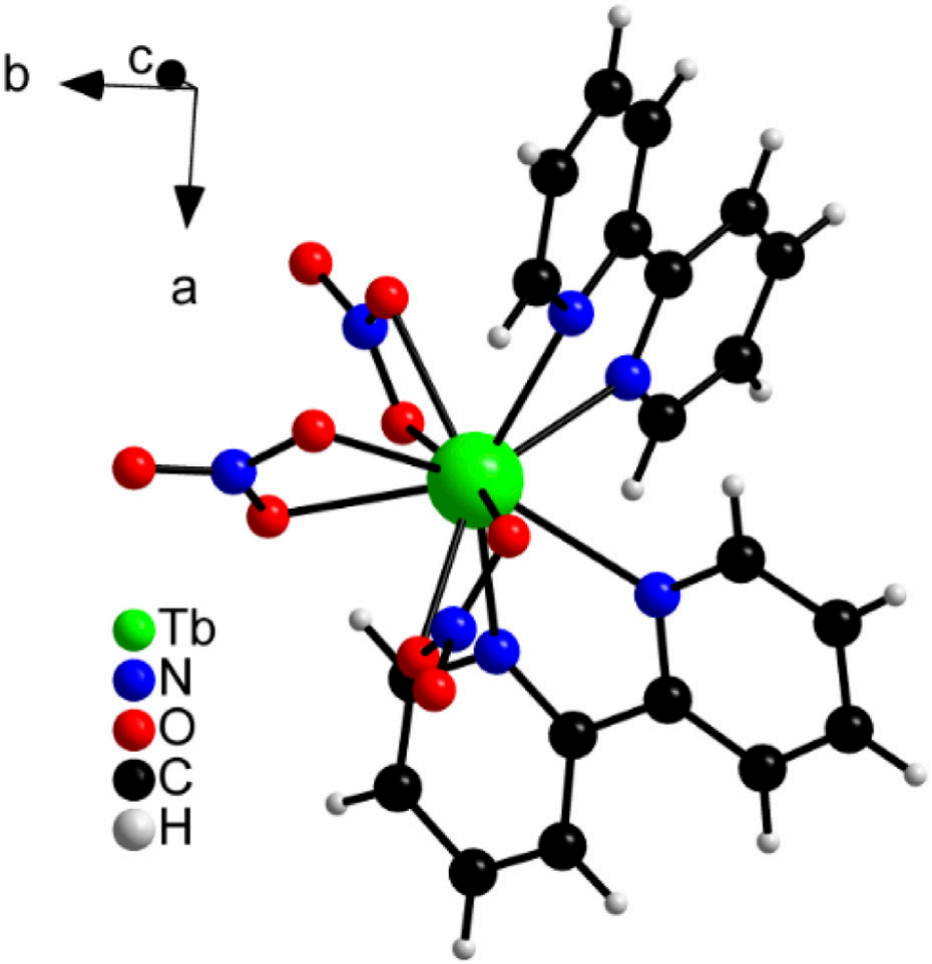
View of the Tb^3+^ coordination in the crystal structure of [Tb (bipy)_2_(NO_3_)_3_].

Moreover, [Tb (bipy)_2_(NO_3_)_3_] composition was confirmed by CHN elemental analysis (Supplementary Table S4). The calculated elemental composition for Tb(C_10_H_8_N_2_)_2_(NO_3_)_3_ was: C, 36.6%; H, 2.5%; N, 14.9%, whereas the elemental composition found showed no significant difference from that calculated and was: C, 36.2%; H, 2.5%; N, 15.0%. In this complex, the central ion Tb^+3^ is coordinated 10-fold, as three nitrate ligands are attached to the metal centre via six oxygen ions and two 2,2'-bipyridine molecules via nitrogen (Figure 3; Supplementary Figure S3). The binding distances between the complex centre and the ligands as well as detailed crystallographic information are listed in Supplementary Tables S5, S6. The lengths correspond to the values known in literature (Moss and Sinha, 1969) and also show good similarity to the values of the [Eu (bipy)_2_(NO_3_)_3_] complex (Supplementary Table S10) (Cotton et al., 2003; Cotton and Raithby, 2017). Supplementary Figure S4 shows also the good agreement of the measured diffraction pattern for Experiment 1 with the calculated pattern resulting from the single crystal structure solution.

Therefore, the structure of [Tb (bipy)_2_(NO_3_)_3_] solved by direct methods from serial crystallography measurements described in Section 3.1 is in good agreement with the structure determined by single crystal measurements.

### 3.3 Optical properties of [Tb (bipy)_2_(NO_3_)_3_]

**FIGURE 4:**
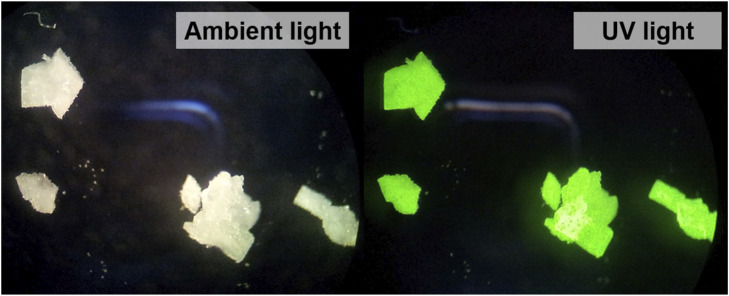
Synthesized [Tb (bipy)_2_(NO_3_)_3_] illuminated with ambient (left) and UV light (right).

**FIGURE 5:**
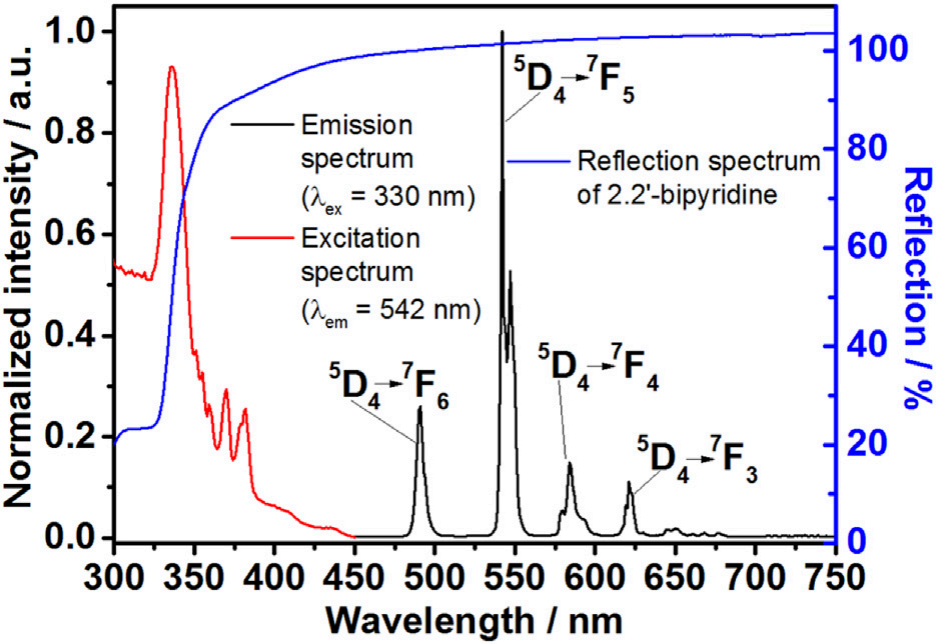
Optical reflectance of 2,2′-bipyridine (blue curve), excitation (λ_em_ = 542 nm, red curve) and emission (λ_ex_ = 330 nm, black curve) spectra of [Tb (bipy)_2_(NO_3_)_3_] crystals.

[Tb (bipy)_2_(NO_3_)_3_] was first synthesized *ex situ* in ethanol, as described in detail in the experimental section (Experiment 1, Table 1). The synthesized crystals were colorless under daylight, and showed green luminescence upon irradiation with UV light (λ_ex_ = 330 nm) (Figure 4). This photoluminescence behavior can be explained by studying the excitation and emission spectra of [Tb (bipy)_2_(NO_3_)_3_] crystals (Figure 5).

The emission spectrum (λ_ex_ = 330 nm) of [Tb (bipy)_2_(NO_3_)_3_] crystals indicates that the characteristic emission peaks due to the ^5^D_4_→ ^7^F_3-6_ electronic transitions are accountable for this compound green luminescence (Ströh et al., 2019). In the emission spectrum, the typical Tb^3+^ emission lines are observed at 491 nm (^5^D_4_ → ^7^F_6_), 542 nm (^5^D_4_ → ^7^F_5_), 589 nm (^5^D_4_ → ^7^F_4_), and 621 nm (^5^D_4_ → ^7^F_3_) (Figure 5, black curve). The excitation spectrum (λ_em_ = 542 nm) of the complex shows a series of excitation bands belonging to the Tb^3+^ and 2,2′-bipyridine electronic transitions at 350–375 and 320–350 nm, respectively (Figure 5, red curve). The reflectance spectrum of pure 2,2′-bipyridine shows that the ligand reflects light strongly in the visible range (400–750) and weakly in the UV region (<350 nm, Figure 3, blue curve). This observation implies that the complex strong absorption in the UV region is a consequence of 2,2′-bipyridine high absorbance in the same region.

The ligand-to-metal energy transfer is detected in the excitation spectrum as a broad strong absorption band with a maximum located at 336 nm (λ_em_ = 542 nm), assigned to the ligand π−π* transition. This transition is also observed in the 2.2′-bipyridine reflectance spectrum as a strong absorption in the UV region (Figure 5, blue curve). In contrast, the electronic transitions of Tb^3+^ ions appear in the excitation spectrum as a series of less intense narrower peaks. This difference in intensities signifies that the ligand-to-metal energy transfer is the primary factor responsible for the [Tb (bipy)_2_(NO_3_)_3_] photoluminescence behavior. Herein; the energy is absorbed by the ligand and transferred to Tb^3+^ ions resulting in metal ion sensitization and emission peaks intensification (Hasegawa et al., 2018). In detail, Zahariev et al. (2021) used density functional theory (DFT)-based approaches to investigate the energy transfer between heterocyclic nitrogen donor chelating antennae and Tb^3+^, revealing the S_0_→S_1_→T_2_→T_1_→^5^D_4_ excitation channel behind the sensitization pathway leading to light emission.

### 3.4 *In situ* monitoring the crystallization process

In this work, rationalization of [Tb (bipy)_2_(NO_3_)_3_] crystallization mechanism was achieved by tailoring its reaction pathway. For this purpose, the influence of the following reaction aspects was investigated: (i) addition rate of the ligand to the metal solution, (ii) initial reactants’ concentrations and (iii) ligand-to-metal ratio.

**FIGURE 6:**
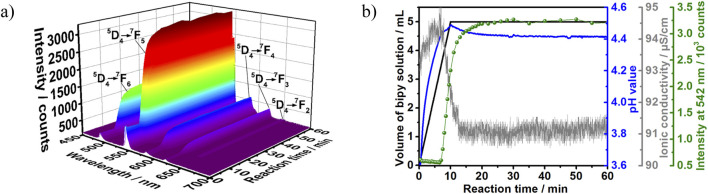
**(A)** 3D representation of the *in situ* luminescence spectra (λ_ex_ = 365 nm) recorded during the synthesis of [Tb (bipy)_2_(NO_3_)_3_] at a ligand addition rate of 0.5 mL/min. **(B)** Respective time-dependence of the addition volume of the 2,2′-bipyridine to Tb^3+^ solutions (black), the pH value (blue), the ionic conductivity (grey) and the normalized emission intensity of the ^5^D_4_→^7^F_5_ Tb^3+^ transition (green) (Exp. 2, Table 1).

Figure 6 shows typical results obtained for monitoring the crystallization of the terbium complex by *in situ* luminescence analysis of coordination sensor (ILACS) (Ruiz Arana et al., 2019), *in situ* measurements of pH value and ionic conductivity (Experiment 2, Table 1). Before beginning the reaction (t < 0 min), *in situ* luminescence spectrum of Tb(NO_3_)_3_·6H_2_O ethanolic solution showed broad low-intensity emission peaks due to the Tb^3+^ electronic transitions within the 4f-shells (Figure 6A). At the time t = 0 min, the splitting behavior and intensities of the emission peaks assigned to the ^5^D_4_→^7^F_6-3_ transitions were governed by the strong quenching effect caused by the protic solvent molecules (Beeby et al., 1999). As discussed in detail by Di Bernardo et al. (2012), trivalent lanthanide ions are hard acids and have a strong affinity for charged ligands or neutral O- and N-donors. During complex formation, ligand coordination to metal ions in solution competes with solvation of the species involved in the reaction, which is highest for cations. Herein, ethanol-Tb^3+^ interaction occurs within the Tb^3+^ coordination sphere, where the luminescence quenching is a consequence of the solvents’ overtone vibrational transitions (Huang et al., 2018). At the early stage of the reaction (t < 7 min), a slight decrease in the luminescence intensity of the ^5^D_4_→^7^F_6-3_ transitions was observed (see Supplementary Figures S5–S7). This decrease is conceivably due to the dilution caused by the increase in the solution volume.

To gain precise insights into the crystallization process, the changes in green emission located at 542 nm was followed, assigned to the ^5^D_4_→^7^F_5_ electronic transition (Manzur et al., 2020; Huang et al., 1999) (Figure 6B, green curve). Upon the ligand solution addition, the luminescence intensity of the ^5^D_4_→^7^F_5_ transition remains nearly constant up to t = 7 min. However, a sharp increase in intensity at t > 7 min was recorded; this observation indicates that the reaction requires approximately 7 min induction time to start the product formation. In detail, at t = 7–12 min and a constant addition rate of the ligand solution, a rapid growth in luminescence intensity was observed. This rise reflects a transformation in the solvation shell surrounding Tb^3+^ ion caused by both the desolvation process and the ligand-to-metal energy transfer process. In this stage, the ligand molecules gradually exchange ethanol molecules in Tb^3+^ local coordination environment, which promotes the increase in the Tb^3+^ ion luminescence during the product formation stage. At t = 12–26 min, the luminescence intensity of the ^5^D_4_→^7^F_5_ transition continued to increase however slowly. At t > 26 min, the emission intensity stabilized indicating the equilibrium state of the reaction and the end of the crystallization. The powdered sample was then washed, dried and subjected to XRD analysis.

To achieve a better understanding of the product formation mechanism, the splitting behavior of the luminescence *peaks* was investigated as well. At the early stage of the reaction (t < 7 min), the recorded luminescence spectrum showed a series of broad asymmetrical emission peaks corresponding to the ^5^D_4_→^7^F_6-3_ transitions of Tb^3+^ ion, these intensities were located in the range from 480 to 530 nm and showed no significant splitting pattern due to their reduced intensity and consequently low spectral resolution (see also Supplementary Figures S5–S7). As the crystallization reaction starts (t > 7 min), the emission peak corresponding to the ^5^D_4_→^7^F_5_ transition (λ_em_ = 542 nm) presented a twofold split and a threefold split was observed for the transition ^5^D_4_→^7^F_4_ (λ_em_ = 583 nm). According to Dalal et al. (2019), there is no pure magnetic dipole allowed transition in the emission spectrum of Tb^3+^. Therefore, the split of the emission peak assigned to ^5^D_4_→^7^F_5_ could be explained by electric dipole contributions within this electronic transition. Changes in the split of the emission peaks assigned to the ^5^D_4_→^7^F_6_ transition are less clear, most likely because of the greater overlap between them due to the high number of sublevels due to the respective high J values. This one-step transformation in the splitting pattern at t = 7 min implies that the Tb^3+^ ion was exposed to two different coordination environments during the course of reaction, namely in the solvation shell and within the product lattice. In this context, it is important to mention that the split pattern observed *in situ* for t > 7 min coincides to the one of the final product recorded *ex situ*, discussed in Figure 5. Therefore, the time range from t = 0–7 min correlates to the replacement of solvent molecules by the ligand in the Tb^3+^ inner sphere and the product was formed under these synthesis conditions via a single-step transformation mechanism. It can be also noticed that the splitting becomes stronger and clearer throughout the reaction. This is due to the change in Tb^3+^ local symmetry during the product formation and the time-dependent increase in the ligand-to-metal energy transfer caused by the increase in the luminescent product concentration (Supplementary Figure S6). Besides the splitting behavior, changes in the intensity ratio (*I*) of the ^5^D_4_→^7^F_5_ (magnetic dipole transition) to the ^5^D_4_→^7^F_6_ (electric dipole transition) can also indicate changes in Tb^3+^ ion local symmetry (Manzur et al., 2020). The forced electric dipole ^5^D_4_→^7^F_6_ transition is forbidden in the free lanthanide ion state (Bao et al., 2018) and its intensity is highly suppressed when Tb^3+^ occupies a high symmetry site (Yin et al., 2017). However, the time-dependent change in the ions coordination sphere during the product formation decreases the local symmetry, which results in an increase in ^5^D_4_→^7^F_6_ peak height. Consequently, the increase in ^5^D_4_→^7^F_6_ transition emission intensity lowered the intensity ratio (*I*) as depicted in Supplementary Figure S7.

Additional deep insights are delivered by *in situ* measurements of the pH values, confirming the data obtained by the ILACS approach discussed above. The steady increase in the pH values at t < 7 min (Figure 6B) indicates a constant increase in the ligand concentration, which evidences the presence of the ligand as free basic bipy molecules in the solution and confirms the induction time before the formation of product. As the definition of pH is based on its measurement in water (Chan and Tan, 2016), the measurements carried out in this work on ethanol are valuable for showing trends due to the evolution of chemical reactions, but should not be considered as isolated absolute values. However, at t = 7–10 min, a deceleration in the pH curve is noticed despite the continuous addition of the ligand solution. Clearly, the shape of the pH curves at this period is a result of two simultaneous effects: the decrease of pH due to the product formation and the increase of the pH value due to the further addition of the solution. This decrease is associated with the ligand molecules consumption from the solution and the incorporation of these basic molecules into the complex formation. At t = 10–26 min, the pH curve steadily decreases then reaches a plateau at t > 26 min, indicating a decrease in the free bipy concentration due to the product formation followed by reaction completion at t > 26 min.

The simultaneously recorded ion conductivity measurement also confirmed the product formation indicated by the ILACS approach. Hence, a significant drop in the ionic conductivity was recorded at t = 7–12 min, indicating the uptake of the charge carriers from the solution due to the beginning of crystallization. Afterward, the ion conductivity curve (Figure 6B) shows continual decrease during the crystal growth stage (t = 10–26 min), followed by plateau at the end of the crystallization time (t > 26 min).

In an additional experiment for confirming the formation of [Tb (bipy)_2_(NO_3_)_3_], samples were collected from the reaction vessel at predetermined times and analyzed using *ex situ* X-ray diffraction analysis (Experiment 2, Supplementary Figure S8). Up to t = 5 min of the reaction time, no solid was formed in the reaction vessel, which is in agreement with the delayed crystallization indicated by the analogue *in situ* luminescence measurements (Figure 6A). Subsequently, further samples were collected in the time range from t = 5–60 min. The *ex situ* XRD pattern of the solid collected at t = 5 min indicates the presence of two crystalline phases, these phases were identified by comparison with the simulated diffraction patterns of the target product, generated by the structure solution reported in Section 3.2, and of the precursor Tb(NO_3_)_3_·6H_2_O (Moret et al., 1990) (Supplementary Figure S8). As the reaction proceeded, the reflections corresponding to the starting material decreased in intensities until t = 7 min and disappeared completely at t = 10 min, while the recorded Bragg reflections at, e.g. t = 12 and 60 min confirmed the product formation as a pure phase.

### 3.5 Influence of addition rate on the crystallization pathway

The influence of ligand addition rate on the crystallization mechanism is an important synthetic parameter to consider. In this work, the crystallization process was studied using the addition rates of 0.5 and 10 mL/min in Experiments 2 and 3, respectively (Table 1). In general, speeding up the addition rate of the ligand solution resulted in a reduced induction time. For instance, at the addition rate 0.5 mL/min, the offset of the luminescence intensity started only at t = 7 min, while under the accelerated addition rate (10 mL/min) this value remarkably shortened to 1 min (Figure 6A; Supplementary Figure S9).

Similar to Experiment 2 (Supplementary Figures S5, S6), the recorded *in situ* luminescence spectra for Experiment 3 showed a direct transformation in ^5^D_4_→^7^F_5_ transition splitting pattern (Supplementary Figure S10). Under the accelerated conditions, the emission intensity evolved from a broad emission peak to a twofold split indicating a direct single-step transformation mechanism between the Tb^3+^ ion in the solution and in the product, without the formation of reaction intermediates under these conditions. Also for this experiment, the *ex situ* X-ray diffraction analysis of samples collected during the synthesis confirmed the direct formation of the product at t = 2 min (Supplementary Figure S11).

The pH and ionic conductivity measurements (Supplementary Figure S12, blue and gray curves, respectively) showed comparable responses toward the accelerated product formation, the curves of these measurements showed similar behavior patterns under the studied addition rates (0.5 and 10 mL/min). However, the response times of these measurements shifted to shorter durations under the accelerated addition rate. To illustrate, under the addition rate 0.5 mL/min, the pH curve reaches a maximum at t = 10 min (Figure 6B), while under the addition rate 10 mL/min, this maximum shifted in time to t = 1.5 min (Supplementary Figure S12), this is due to fast increase in the ligand concentration in the solution.

Likewise, the *in situ* ionic conductivity measurement showed an analogous behavior. The drastic reduction in the ionic conductivity shifted in time from 7 min to 45 s upon changing the flow rate from 0.5 (Figure 6B) to 10 mL/min (Supplementary Figure S12), respectively. This also indicates that the accelerated addition rate results in an early uptake of the charge carriers from the solution and an early product formation. Building on these observations, it can be stated that the flow rate has a strong influence over the crystallization kinetics but less impact on the crystallization mechanism.

### 3.6 Influence of solution concentration on crystallization pathway and intermediate formation: comparison to synchrotron-based *in situ* XRD measurements

*Ex situ* XRD measurements are helpful for offering insights into the crystal structure of the formed compounds. However, information extracted from this approach was acquired for products that have been subjected to quenching, centrifuging and drying, which makes this analysis useful as a preliminary approach to analyze the crystallization duration but inadequate representative of the overtime structural evolution. Therefore, *in situ* synchrotron X-ray diffraction has been utilized for elucidating the stages of transformation occurring in the reactor under different reaction conditions and to explicitly avoid *ex situ* structural changes caused by the isolation and drying steps.

The findings obtained from our previous reported study on the crystallization process of [Eu (bipy)_2_(NO_3_)_3_] demonstrated that the formation of an intermediate phase depends strongly on the reactants’ concentrations (Polzin et al., 2018). This *in situ* study revealed that [Eu (bipy)_2_(NO_3_)_3_] crystallizes directly under diluted reaction conditions. On the other hand, an unknown crystalline intermediate was detected under high concentration conditions. This observed species was highly reactive and transformed completely to the target product via a solid-solid transformation mechanism during the reaction. Although a direct comparison between the stability of [Eu (bipy)_2_(NO_3_)_3_] and [Tb (bipy)_2_(NO_3_)_3_] is not possible due to the different experimental conditions used in the present and in the previous (Polzin et al., 2018) publications, Eu^3+^ complexes are generally expected to be slightly more labile than the Tb^3+^ ones. This fact is explained, for example, by the smaller ionic radius of Tb^3+^ and the increase in ion charge density (Raj, 2014). In the present article, time-resolved luminescence investigations of [Tb (bipy)_2_(NO_3_)_3_] formation revealed a single-step crystallization mechanism under the diluted conditions (Experiment 2, Table 1). Therefore, the influence of increasing the reactants’ concentrations to 300% (Experiment 4, Table 1) was consecutively studied here to evaluate the possibility of multistep crystallization pathway under high concentration conditions. Also, for the Tb^3+^ analog complex, the formation of [Tb (bipy)_2_(NO_3_)_3_] under the tested conditions (300%, Experiment 4, Table 1) was followed by ILACS and synchrotron X-ray diffraction analysis at PETRA III (P08 beamline). Simultaneously, variations in the solution turbidity were tracked monitoring changes in the *in situ* light transmission measurements. For these measurements, the used light source was positioned outside the reaction vessel while its intensity was recorded through an optical fiber immersed inside the vessel.

**FIGURE 7:**
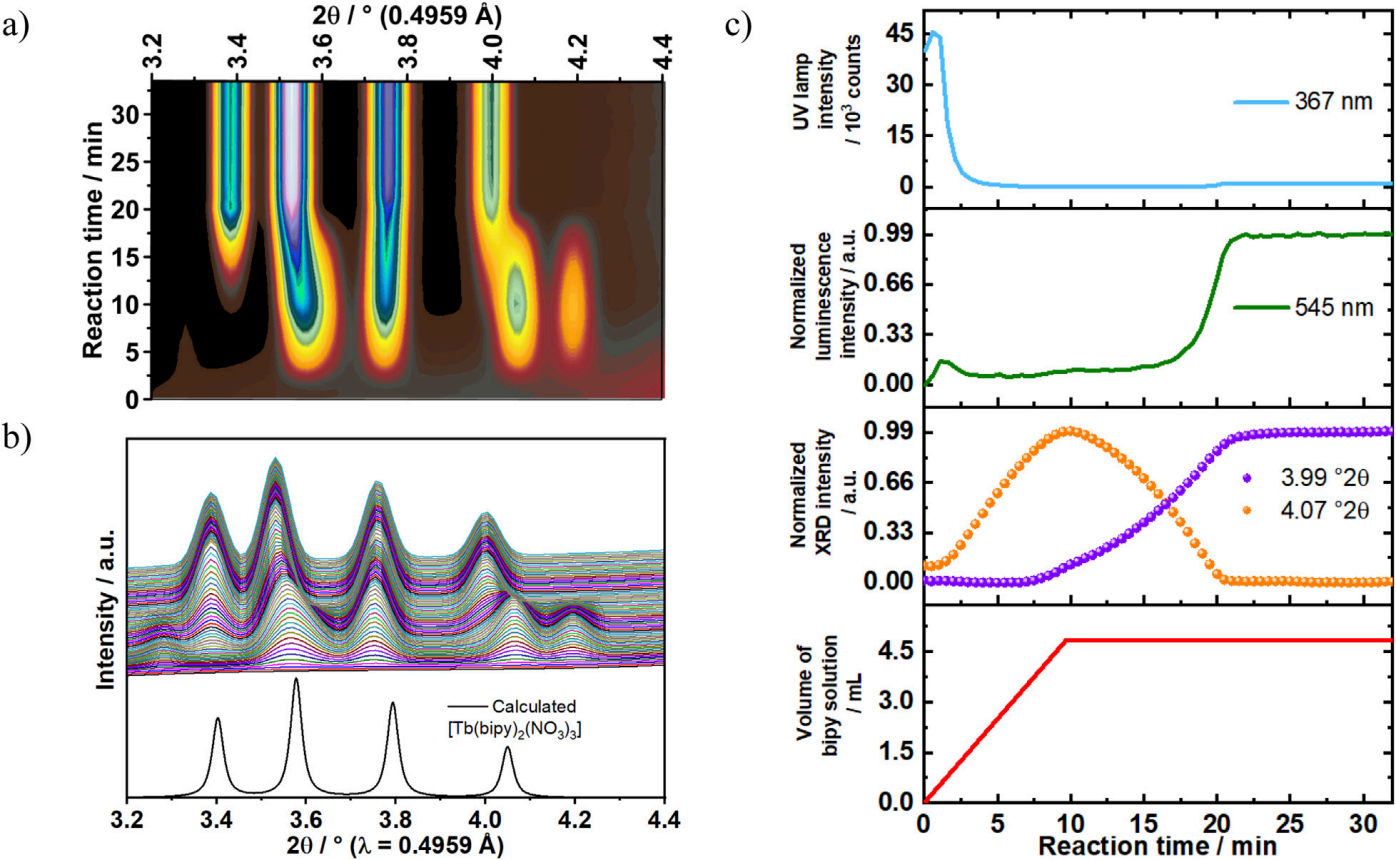
**(A)** *In situ* XRD patterns recorded at the P08 DESY beamline (λ = 0.4959 Å) are **(B)** compared to calculated diffraction patterns for [Tb (bipy)_2_(NO_3_)_3_] (ratio bipy:Tb = 2:1, Exp. 4). **(C)** Time-dependence of light transmission intensity at 367 nm (blue curve) and emission intensity of the Tb^3+ 5^D_4_→^7^F_4_ transition at 545 nm (green curve) are compared to simultaneous *in situ* XRD measurements at, e.g. 3.99° 2θ {violet doted curve, [Tb (bipy)_2_(NO_3_)_3_]} and 4.07°2θ (orange doted curve, intermediate) as well as the addition rate of the bipy solution to the reactor containing terbium (III) nitrate at 0.5 mL/min (red curve).

The ILACS characterization method offers the advantage of detecting luminescent species independent if they are dissolved, amorphous or crystalline. This feature was beneficial in our study of crystallization pathways in both diluted and concentrated systems in Experiments 2 and 4, respectively. Increasing the reactants’ concentration by 300% in Experiment 4 (Table 1) showed an impact over the luminescence onset time (Figure 7). As mentioned previously, under the diluted reaction conditions in Experiment 2 (100% reactants’ concentration, Table 1), 7 min were necessary for the ^5^D_4_→^7^F_5_ emission peak to exhibit a sharp increase in intensity. On the other hand, the threefold increase in concentration in Experiment 4 revealed a different time-dependent luminescence behavior. At t = 0–1.5 min, a slight increase in the ^5^D_4_→^7^F_5_ emission intensity was detected and kept constant until approximately t = 17 min (Figure 7). As the reaction proceeded, the intensity remarkably increased in the time range of t = 17–21 min showing two distinct growth rates for this reaction, indicating the formation of two different compounds.

**FIGURE 8:**
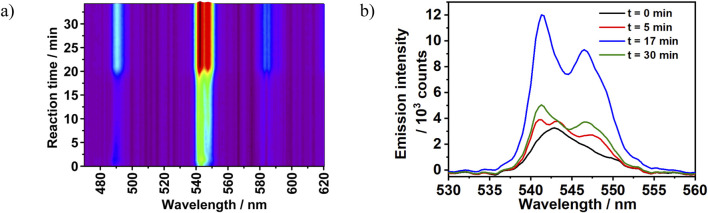
**(A)** *In situ* emission intensity (λ_ex_ = 365 nm) recorded during the synthesis of [Tb (bipy)_2_(NO_3_)_3_] at 300% reactant concentration with the addition rate of 2,2′-bipyridine at 0.5 mL/min (Experiment 4). **(B)** Time-dependent changes in ^5^D_4_→^7^F_5_ splitting pattern recorded during the synthesis of [Tb (bipy)_2_(NO_3_)_3_] (ratio bipy:Tb^3+^ = 2:1, Experiment 4).

This effect of reactant concentration on the crystallization pathway can be also observed on the ^5^D_4_→^7^F_5_ transition splitting behavior. The reaction conducted under diluted reactants condition (100% reactants’ concentration, Experiment 2) showed a direct transformation from a single broad emission intensity to twofold split (^5^D_4_→^7^F_5_ transition). However, the ^5^D_4_→^7^F_5_ transition emission peaks recorded under the concentrated conditions (Experiment 4) showed a different time-dependent transition splitting behavior. As shown in Figure 8, the ^5^D_4_→^7^F_5_ transition peak was recorded in the range of λ_em_ = 537–553 nm with a maximum located at 542 nm. At the beginning of the reaction (t = 0 min), this intensity had a broad asymmetrical appearance with no observable split. As the reaction progressed, the emission intensity developed three peaks splitting pattern at t = 5 min. This pattern evolved afterwards into two peaks at t = 17 min and exhibited continuous intensity growth up to the end of reaction period. Interestingly, the observed three peaks splitting under the concentrated reaction conditions confirms a two-step crystallization mechanism. In the first step an unknown luminescent phase is formed with three-folds split, which therefore transformed to [Tb (bipy)_2_(NO_3_)_3_] in the second step. Unfortunately, isolating this phase by quenching experiments for *ex situ* characterization was not possible due to its low stability, also showing the importance of *in situ* analysis techniques.

Besides the ILACS results, synchrotron-based *in situ* XRD and solution turbidity measurements also revealed the intermediate formation. During the first 2 min of Experiment 4, an increase in the Bragg reflection at a scattering angle of 2θ = 4.07° was detected by *in situ* XRD measurement. This reflection reached a maximum intensity at t = 10 min, followed by a complete decay at t = 21 min (Figure 7A). This behavior indicates the formation of an unknown crystalline intermediate during the reaction. This period was also assigned by ILACS measurement as the three-fold splitting pattern stage. However, despite of the increase in the Bragg reflection corresponding to the intermediate in the described period, no noticeable increase in the ^5^D_4_→^7^F_5_ transition emission intensity at 545 nm was observed. It implies that the generated intermediate is weakly luminescent species and that it has a greater impact on the ^5^D_4_→^7^F_5_ transition emission-splitting pattern (Figures 7, 8) than on the emission intensity. As the reaction proceeded, an increase in the Bragg reflection at 2θ = 3.99° was recorded at t = 7 min. This reflection reached a plateau at t = 21 min, showing an equilibrium state and the formation of a stable crystalline phase. This phase was identified as [Tb (bipy)_2_(NO_3_)_3_] by comparing the *in situ* XRD patterns to the calculated diffraction patterns obtained for [Tb (bipy)_2_(NO_3_)_3_] (Section 3.2. The time-dependent evolution of the Bragg reflection at 2θ = 3.99° follows a sigmoidal growth model, in which the crystalline intermediate is generated at the early stage of the reaction and then crystallizes into the product [Tb (bipy)_2_(NO_3_)_3_]. This model of transformation which is frequently observed in solid-state transformations (Pienack et al., 2009), additionally supports the two-step transformation mechanism indicated by the ^5^D_4_→^7^F_5_ transition emission-splitting behavior recorded by ILACS measurement. As the reflections correlated to the reaction intermediate reach complete decays, the reflections assigned to the product reach their maximums values, which confirms the conversion of the intermediate to product through a solid-solid transformation.

Additional results obtained by *in situ* monitoring changes in turbidity (Pienack et al., 2016) were found to be consistent with the result obtained by ILACS and XRD measurements (Figure 7). At t = 0–2 min, a slight increase in the light transmission was observed, this increase indicates a decrease in the solution turbidity caused by an initial dilution. As the reaction progresses, a strong decrease in the in the light transmission was recorded at the time range t = 2–3 min, demonstrating an increase in the solution turbidity and the beginning of solid compounds. It is worth noting that this time range (t = 2–3 min) associates with the induction time detected by *in situ* XRD measurement. At t = 3 min, the turbidity strongly increases, indicating the formation of the reaction intermediate, slightly decreasing upon its disappearance at t = 21 min.

### 3.7 Influence of ligand-to-metal ratio on crystallization pathway

**FIGURE 9:**
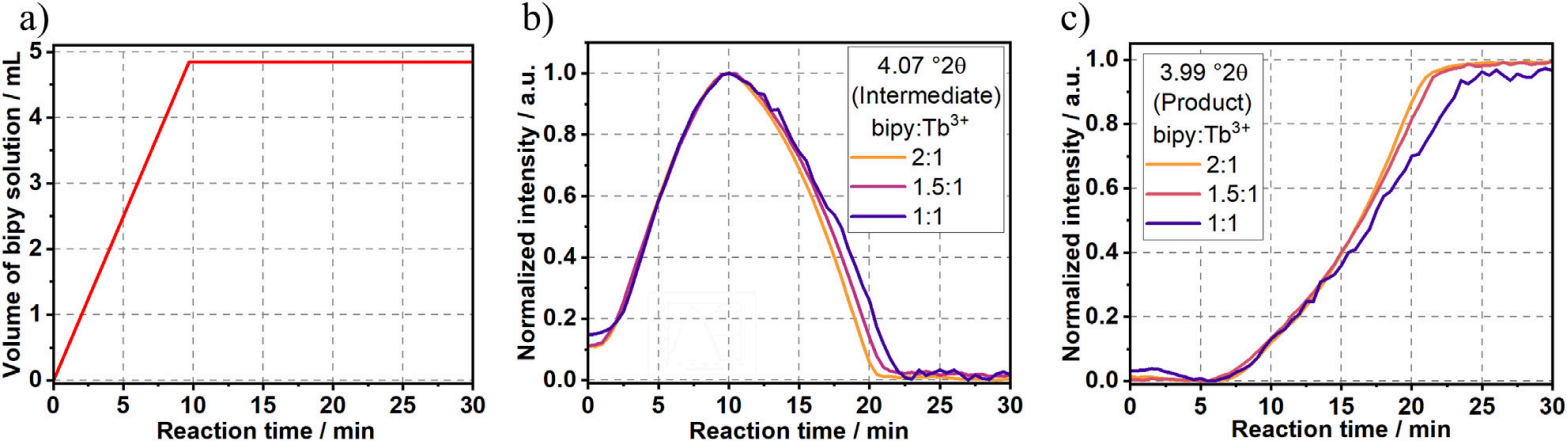
Time dependence of **(A)** the addition of bipy to the Tb^3+^ solution as well as intensity of the synchrotron-based Bragg peak assigned to **(B)** the intermediate at 4.07°2θ and to **(C)** the [Tb (bipy)_2_(NO_3_)_3_] product at 3.99°2θ.

Since increasing the reactants’ concentrations significantly influenced [Tb (bipy)_2_(NO_3_)_3_] crystallization pathways (Experiment 2 and 4), additional experiments were conducted in order to investigate the influence of the ligand-to-metal ratio (bipy:Tb^3+^) on the crystallization mechanism. In Experiments 5 and 6 (Table 1), the reaction was conducted using bipy:Tb^3+^ molar ratios of, respectively, 1.5:1 (Supplementary Figures S13–S15) and 1:1 (Supplementary Figure S16–S18). As shown in Figure 9, reducing the bipy:Tb^3+^ ratio below to the stoichiometric one necessary for the [Tb (bipy)_2_(NO_3_)_3_] formation (2:1), systematically delayed the formation of the product and prolonged the existence of the intermediate.

## 4 Conclusion

The structure of [Tb (bipy)_2_(NO_3_)_3_] solved from serial crystallography is comparable to the one obtained via single-crystal measurements, giving a further demonstration of the feasibility of this approach for solving structures of inorganic-organic hybrid compounds with crystals size down to the nanoscale, using nanofocused beams. The present study found that it was necessary to merge data from 288,000 crystals for *ab initio* solution of carbon, nitrogen and oxygen atoms in the structure. This motivates the development of high-repetition-rate and high-X-ray-energy facilities to realize *in situ* serial crystallography studies to determine the structure of intermediate phases formed during crystallization of such fluorescent complexes and other inorganic compounds. Hence, *in situ* monitoring of reactions pathways provided real-time, high-quality information on phase-phase transformations during the reaction. In this work, we reported a detailed study on [Tb (bipy)_2_(NO_3_)_3_] crystallization process using experimental characterization, spectroscopic and crystallographic methods. Investigating the time-dependent changes in reaction dynamics was achieved by combining information extracted from *in situ* luminescence analysis of coordination sensors (ILACS), *in situ* synchrotron X-ray diffraction, *ex situ* luminescence and X-ray diffraction, in addition to pH, solution turbidity and ion conductivity measurements. Additionally, the influence of synthesis parameters on the luminescent complex crystallization pathways was studied. The *crystallization* behavior was found to be primarily *dependent* on the reactant’s concentrations; under high concentrations, reactions proceed via a crystalline intermediate formation, whereas no intermediate was formed under low concentrations. Changing the concentrations also influenced the reaction induction time and the evolution of emission splitting patterns. The crystallization reaction was also influenced by the ligand-to-metal molar ratios. Reducing the ligand-to-metal ratios below 2:1 systematically delayed the product formation and prolonged the intermediate lifetime. These *in situ* results are extremely important since they open doors for controlling the formation of the intermediate, enabling its possible isolation and solution of its crystal structure in the future.

## Data Availability

The original contributions presented in the study are included in the article/Supplementary Material, further inquiries can be directed to the corresponding authors. CCDC-2406109 (single crystal) and CCDC-2427847 (serial crystallography) contain the supplementary crystallographic data of [Tb(bipy)2(NO3)3]. These data can be obtained free charge from the Cambridge Crystallographic Data Centre via http://www.ccdc.cam.ac.uk/data_request/cif.
