# Supplementary material for: In situ monitoring of ligand-to-metal energy transfer in combination with synchrotron-based X-ray diffraction methods to elucidate the synthesis mechanism and structural evolution of lanthanide complexes
Source: Front Chem. 2025 Apr 17;13:1536383. doi: 10.3389/fchem.2025.1536383 (PMC12044340; doi:10.3389/fchem.2025.1536383)

checkCIF/PLATON report

Structure factors have been supplied for datablock(s) pp211a

THIS REPORT IS FOR GUIDANCE ONLY. IF USED AS PART OF A REVIEW PROCEDURE FOR PUBLICATION, IT SHOULD NOT REPLACE THE EXPERTISE OF AN EXPERIENCED CRYSTALLOGRAPHIC REFEREE.

No syntax errors found.   CIF dictionary   Interpreting this report

Datablock: pp211a

```
Bond precision:   C-C = 0.0048 A           Wavelength=0.71073

Cell:              a=16.7014(5)      b=9.0291(2)      c=14.9826(4)
                   alpha=90          beta=90          gamma=90
Temperature:       170 K

                 Calculated                  Reported
Volume           2259.36(10)                 2259.36(10)
Space group      P b c n                     P b c n
Hall group       -P 2n 2ab                   -P 2n 2ab
Moiety formula   C20 H16 N7 O9 Tb            ?
Sum formula      C20 H16 N7 O9 Tb            C20 H16 N7 O9 Tb
Mr               657.33                      657.32
Dx,g cm-3        1.933                       1.932
Z                4                           4
Mu (mm-1)        3.199                       3.199
F000             1288.0                      1288.0
F000'            1287.88
h,k,lmax         21,11,19                    21,11,19
Nref             2474                        2474
Tmin,Tmax        0.743,0.825                 0.537,0.719
Tmin'            0.674

Correction method= # Reported T Limits: Tmin=0.537 Tmax=0.719
AbsCorr = NUMERICAL

Data completeness= 1.000            Theta(max)= 27.003

                                                     wR2(reflections)=
R(reflections)= 0.0338( 2112)                        0.0921( 2474)

S = 1.127                  Npar= 170
```

The following ALERTS were generated. Each ALERT has the format
test-name_ALERT_alert-type_alert-level.
Click on the hyperlinks for more details of the test.

Alert level C

```
PLAT242_ALERT_2_C Low    'MainMol' Ueq as Compared to Neighbors of        N2 Check
PLAT906_ALERT_3_C Large K Value in the Analysis of Variance ......      7.335 Check
```

Alert level G

```
PLAT232_ALERT_2_G Hirshfeld Test Diff (M-X)  Tb1      --O1       .        5.5 s.u.
PLAT232_ALERT_2_G Hirshfeld Test Diff (M-X)  Tb1      --O2       .        5.6 s.u.
PLAT432_ALERT_2_G Short Inter X...Y Contact  O5       ..C20      .       2.99 Ang.
                                              1-x,1+y,1/2-z  =      3_665 Check
PLAT432_ALERT_2_G Short Inter X...Y Contact  O5       ..C20      .       2.99 Ang.
                                                    x,1+y,z  =      1_565 Check
PLAT764_ALERT_4_G Overcomplete CIF Bond List Detected (Rep/Expd) .       1.11 Ratio
PLAT794_ALERT_5_G Tentative Bond Valency for Tb1       (III)     .       3.81 Info
PLAT883_ALERT_1_G Absent Datum for _atom_sites_solution_primary ..     Please Do !
PLAT969_ALERT_5_G The 'Henn et al.' R-Factor-gap value ..........      6.391 Note
              Predicted wR2: Based on SigI**2  1.44 or SHELX Weight  8.17
PLAT978_ALERT_2_G Number C-C Bonds with Positive Residual Density.          0 Info
```

0 **ALERT level A** = Most likely a serious problem - resolve or explain
0 **ALERT level B** = A potentially serious problem, consider carefully
2 **ALERT level C** = Check. Ensure it is not caused by an omission or oversight
9 **ALERT level G** = General information/check it is not something unexpected

1 ALERT type 1 CIF construction/syntax error, inconsistent or missing data
6 ALERT type 2 Indicator that the structure model may be wrong or deficient
1 ALERT type 3 Indicator that the structure quality may be low
1 ALERT type 4 Improvement, methodology, query or suggestion
2 ALERT type 5 Informative message, check

It is advisable to attempt to resolve as many as possible of the alerts in all categories. Often the minor alerts point to easily fixed oversights, errors and omissions in your CIF or refinement strategy, so attention to these fine details can be worthwhile. In order to resolve some of the more serious problems it may be necessary to carry out additional measurements or structure refinements. However, the purpose of your study may justify the reported deviations and the more serious of these should normally be commented upon in the discussion or experimental section of a paper or in the "special_details" fields of the CIF. checkCIF was carefully designed to identify outliers and unusual parameters, but every test has its limitations and alerts that are not important in a particular case may appear. Conversely, the absence of alerts does not guarantee there are no aspects of the results needing attention. It is up to the individual to critically assess their own results and, if necessary, seek expert advice.

Publication of your CIF in IUCr journals

A basic structural check has been run on your CIF. These basic checks will be run on all CIFs submitted for publication in IUCr journals (*Acta Crystallographica*, *Journal of Applied Crystallography*, *Journal of Synchrotron Radiation*); however, if you intend to submit to *Acta Crystallographica Section C* or *E* or *IUCrData*, you should make sure that full publication checks are run on the final version of your CIF prior to submission.

Publication of your CIF in other journals

Please refer to the *Notes for Authors* of the relevant journal for any special instructions relating to CIF submission.

PLATON version of 11/11/2024; check.def file version of 11/11/2024

Datablock pp211a - ellipsoid plot

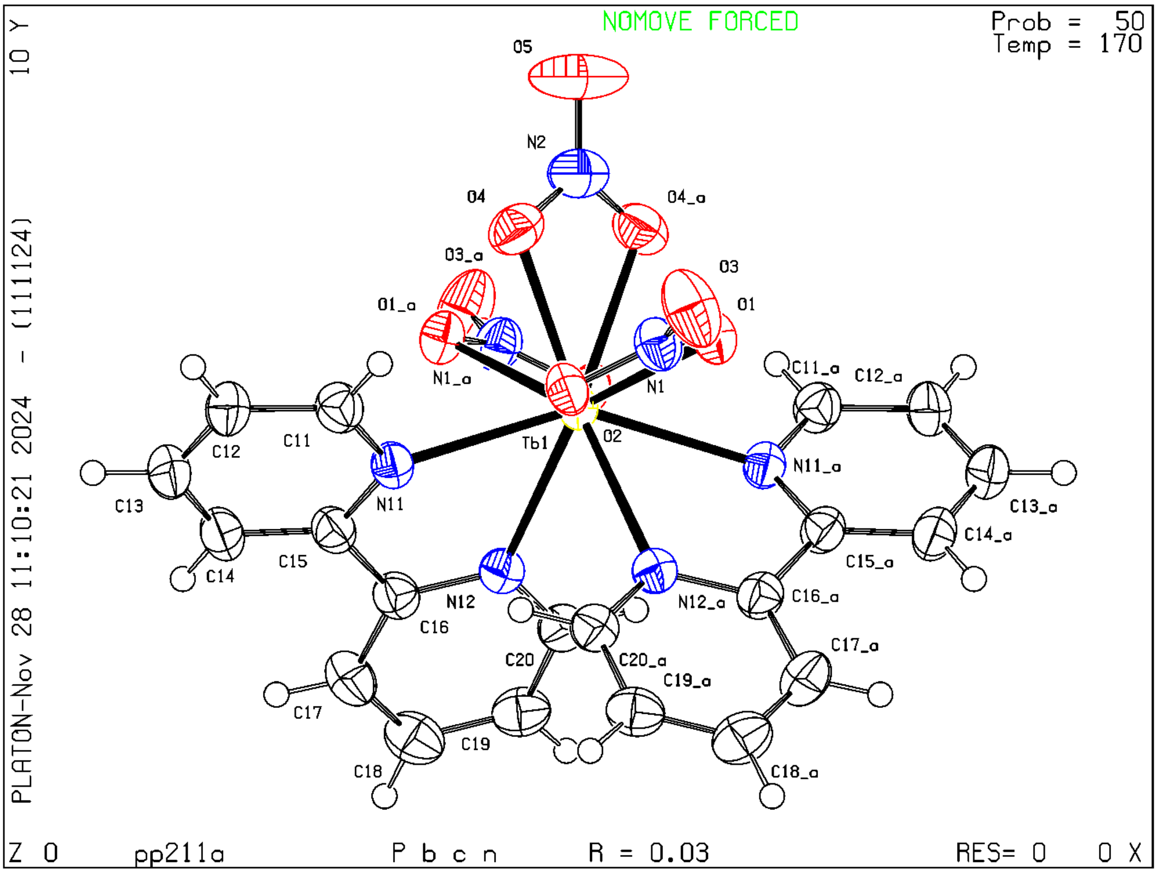

Supplement: Supplementary file 1 [file DataSheet2.pdf]
