# Supplementary material for: In situ monitoring of ligand-to-metal energy transfer in combination with synchrotron-based X-ray diffraction methods to elucidate the synthesis mechanism and structural evolution of lanthanide complexes
Source: Front Chem. 2025 Apr 17;13:1536383. doi: 10.3389/fchem.2025.1536383 (PMC12044340; doi:10.3389/fchem.2025.1536383)
Supplement: Supplementary file 2 [file DataSheet1.pdf]

Supplementary Material

In situ monitoring of ligand-to-metal energy transfer in combination with synchrotron-based X-ray diffraction methods to elucidate the synthesis mechanism and structural evolution of lanthanide complexes

Ban H. Al-Tayyem,[1] Philipp Müscher-Polzin,[1] Kanupriya Pande, [2, 3] Oleksandr Yefanov, [2, 3] Valerio Mariani, [2, 3] Anja Burkhardt, [2, 3] Henry N. Chapman, [2, 3] Christian Näther, [1] Michael Braun, [1] Marvin Radke, [1] Steve Waitschat, [1] Kenneth R. Beyerlein,* [2, 3]†, Huayna Terraschke*[1]

[1] Institut für Anorganische Chemie, Christian-Albrechts-Universität zu Kiel, Max-Eyth-Str. 2, 24118 Kiel, Germany

[2] Center for Free Electron Laser Science, 22761 Hamburg, Germany.

[3] Deutsches Elektronen-Synchrotron DESY, 22607 Hamburg, Germany

† Current Address: Institut national de la recherche scientifique, Centre Énergie Matériaux Télécommunications, 1650 blvd Lionel-Boulet, Varennes, Québec, Canada

* Correspondence:

Kenneth Beyerlein: kenneth.beyerlein@inrs.ca

Huayna Terraschke: hterraschke@ac.uni-kiel.de

1. Supplementary details to the experimental setup 2
2. Results for *ex situ* synthesis of [Tb(bipy)$_2$(NO$_3$)$_3$] 3
3. *In situ* monitoring the crystallization process 9
   3.1. Solution addition at 0.5 mL/min 9
   3.2. Solution addition at 10 mL/min 11
4. Simultaneous *in situ* luminescence and powder X-ray diffraction methods 13

4.1. Bipy:Tb^{3+} ratio 1.5:1 13

4.2. Bipy:Tb^{3+} ratio 1:1 14

1. Supplementary details to the experimental setup

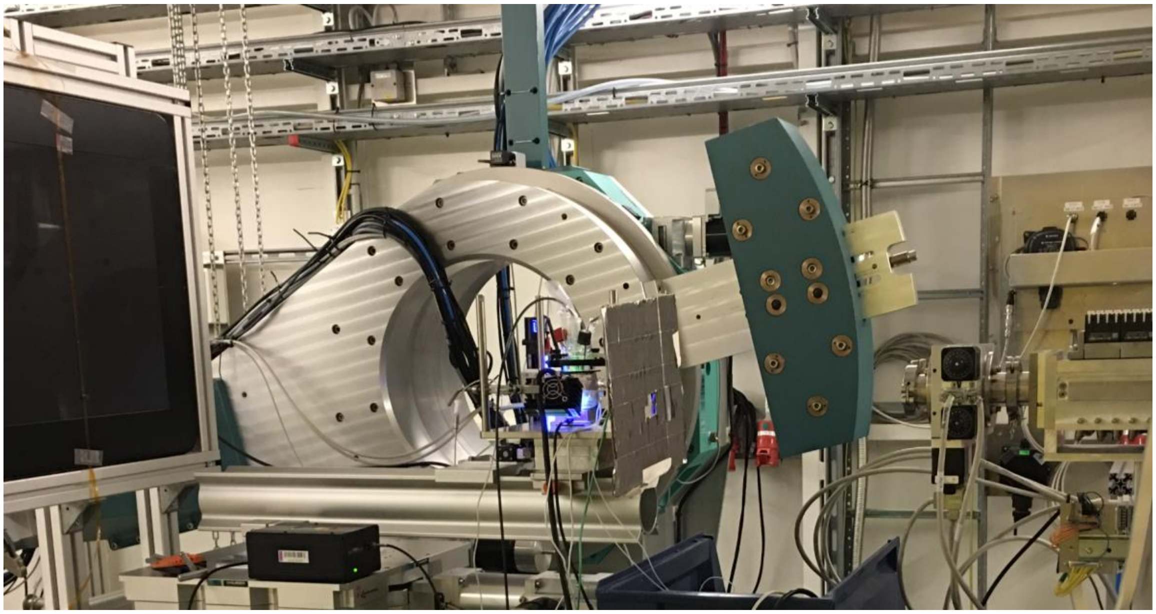

Figure S1: Experimental setup for combining ILACS with *in situ* X-ray diffraction analysis (XRD) at the beamline P08 at DESY.

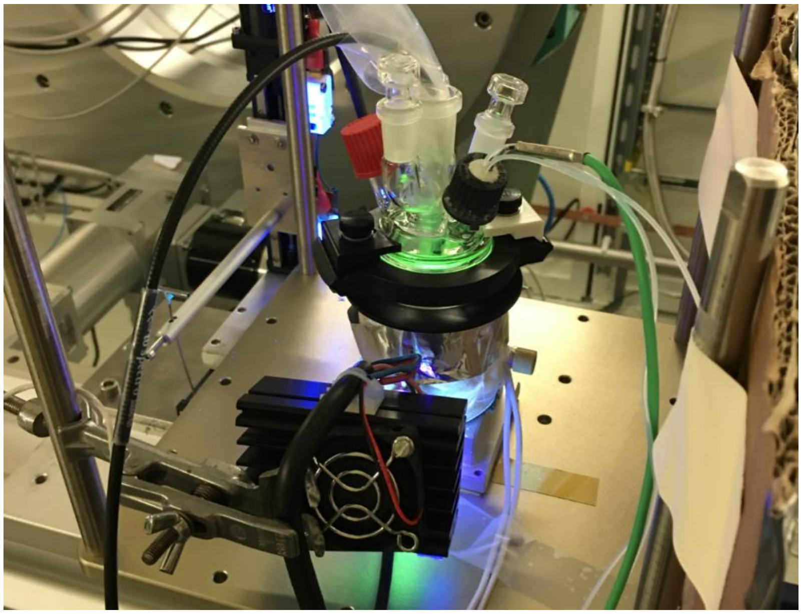

Figure S2: *In situ* reactor containing $[Tb(bipy)_2(NO_3)_3]$ irradiated with UV light at 365 nm.

2. Results for *ex situ* synthesis of [Tb(bipy)$_2$(NO$_3$)$_3$]

Table S1: Selected crystal data and details of the structure determination of [Tb(bipy)$_2$(NO$_3$)$_3$].

Empirical formula	$C_{20}H_{16}N_7O_9Tb$	
Formula weight	657.32	
Temperature	170(2) K	
Wavelength	0.71073 Å	
Crystal system	Orthorhombic	
Space group	*Pbcn*	
Unit cell dimensions	a = 16.7014(5) Å	α = 90°.
	b = 9.0291(2) Å	β= 90°.
	c = 14.9826(4) Å	γ = 90°.
Volume	2259.36(10) Å^3	
Z	4	
Density (calculated)	1.932 Mg/m^3	
Absorption coefficient	3.199 mm^{-1}	
F(000)	1288	
Crystal size	0.08 x 0.10 x 0.11 mm^3	
Theta range for data collection	2.439 to 27.003°.	
Index ranges	-21<=h<=21, -11<=k<=11, -19<=l<=17	
Reflections collected	22336	
Independent reflections	2474 [R(int) = 0.0331]	
Completeness to theta = 25.242°	100.0 %	
Refinement method	Full-matrix least-squares on F^2	
Data / restraints / parameters	2474 / 0 / 170	
Goodness-of-fit on F^2	1.127	
Final R indices [I>2sigma(I)]	R1 = 0.0338, wR2 = 0.0870	
R indices (all data)	R1 = 0.0408, wR2 = 0.0921	
Extinction coefficient	0.0013(3)	
Largest diff. peak and hole	0.834 and -1.019 e.Å^{-3}	

A numerical absorption correction was performed (Tmin/max: 0.5372/0.7187). All non-hydrogen atoms were refined anisotropic. The C-H H atoms were positioned with idealized geometry and refined isotropic with $U_{iso}(H) = 1.2\ U_{eq}(C)$ using a riding model.

Table S2: Atomic coordinates (x 10^4) and equivalent isotropic displacement parameters ($Å^2$x 10^3). U(eq) is defined as one third of the trace of the orthogonalized U^{ij} tensor.

	x	y	z	U(eq)
Tb(1)	5000	5936(1)	2500	30(1)
N(1)	5868(2)	6814(3)	4120(2)	40(1)
O(1)	6165(1)	6906(2)	3334(1)	39(1)
O(2)	5192(2)	6195(3)	4182(2)	38(1)
O(3)	6223(2)	7306(3)	4767(2)	65(1)
N(2)	5000	9161(5)	2500	48(2)
O(4)	4627(2)	8396(3)	3086(2)	48(1)
O(5)	5000	10490(6)	2500	88(2)
N(11)	3717(1)	5151(3)	3255(2)	34(1)
N(12)	4315(2)	3698(3)	1844(2)	33(1)
C(11)	3429(2)	5942(3)	3939(2)	39(1)
C(12)	2627(2)	5925(3)	4190(3)	44(1)
C(13)	2106(2)	5059(4)	3712(2)	45(1)
C(14)	2393(2)	4206(3)	3017(3)	42(1)
C(15)	3201(2)	4262(3)	2805(3)	35(1)
C(16)	3556(2)	3360(3)	2077(2)	36(1)
C(17)	3145(2)	2220(4)	1664(2)	46(1)
C(18)	3517(2)	1402(4)	1001(2)	50(1)
C(19)	4284(2)	1761(3)	756(2)	45(1)
C(20)	4663(2)	2914(3)	1189(2)	39(1)

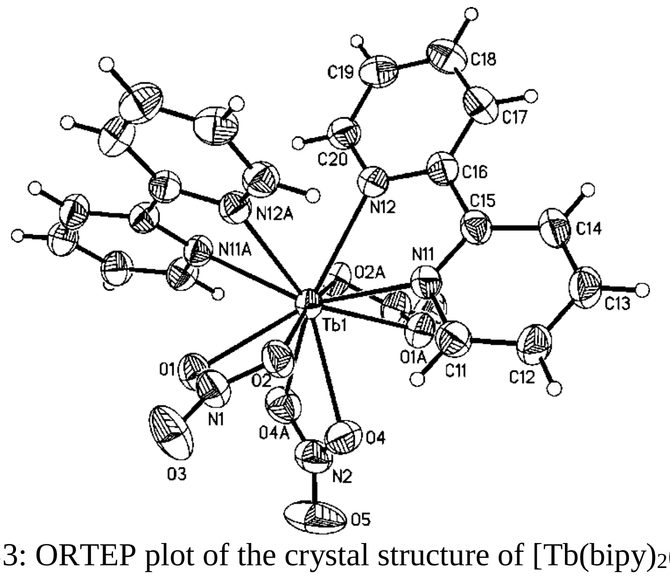

Figure S3: ORTEP plot of the crystal structure of $[Tb(bipy)_2(NO_3)_3]$

Table S3: Bond lengths [Å] and angles [°].

Tb(1)-O(4)	2.469(2)	Tb(1)-N(11)	2.524(2)
Tb(1)-O(4)#1	2.469(2)	Tb(1)-N(11)#1	2.524(2)
Tb(1)-O(1)#1	2.472(2)	Tb(1)-O(2)	2.551(3)
Tb(1)-O(1)	2.472(2)	Tb(1)-O(2)#1	2.551(3)
Tb(1)-N(12)#1	2.521(2)	Tb(1)-N(2)	2.912(4)
Tb(1)-N(12)	2.521(2)	Tb(1)-N(1)#1	2.936(3)
O(4)-Tb(1)-O(4)#1	51.71(12)	N(12)-Tb(1)-O(2)	121.02(8)
O(4)-Tb(1)-O(1)#1	70.26(7)	N(11)-Tb(1)-O(2)	71.92(8)
O(4)#1-Tb(1)-O(1)#1	72.56(8)	N(11)#1-Tb(1)-O(2)	111.20(8)
O(4)-Tb(1)-O(1)	72.56(8)	O(4)-Tb(1)-O(2)#1	103.72(8)
O(4)#1-Tb(1)-O(1)	70.26(7)	O(4)#1-Tb(1)-O(2)#1	66.30(8)
O(1)#1-Tb(1)-O(1)	138.50(10)	O(1)#1-Tb(1)-O(2)#1	50.91(7)
O(4)-Tb(1)-N(12)#1	134.20(9)	O(1)-Tb(1)-O(2)#1	124.45(7)
O(4)#1-Tb(1)-N(12)#1	138.19(8)	N(12)#1-Tb(1)-O(2)#1	121.03(8)
O(1)#1-Tb(1)-N(12)#1	146.90(7)	N(12)-Tb(1)-O(2)#1	68.37(8)
O(1)-Tb(1)-N(12)#1	74.34(7)	N(11)-Tb(1)-O(2)#1	111.20(8)
O(4)-Tb(1)-N(12)	138.19(8)	N(11)#1-Tb(1)-O(2)#1	71.93(8)
O(4)#1-Tb(1)-N(12)	134.20(9)	O(2)-Tb(1)-O(2)#1	169.49(11)
O(1)#1-Tb(1)-N(12)	74.34(7)	O(4)-Tb(1)-N(2)	25.86(6)
O(1)-Tb(1)-N(12)	146.90(7)	O(4)#1-Tb(1)-N(2)	25.86(6)
N(12)#1-Tb(1)-N(12)	73.45(11)	O(1)#1-Tb(1)-N(2)	69.25(5)
O(4)-Tb(1)-N(11)	83.05(8)	O(1)-Tb(1)-N(2)	69.25(5)
O(4)#1-Tb(1)-N(11)	128.78(8)	N(12)#1-Tb(1)-N(2)	143.27(6)
O(1)#1-Tb(1)-N(11)	70.00(8)	N(12)-Tb(1)-N(2)	143.27(6)
O(1)-Tb(1)-N(11)	122.74(8)	N(11)-Tb(1)-N(2)	106.30(5)
N(12)#1-Tb(1)-N(11)	89.16(8)	N(11)#1-Tb(1)-N(2)	106.30(5)
N(12)-Tb(1)-N(11)	64.20(8)	O(2)-Tb(1)-N(2)	84.75(5)
O(4)-Tb(1)-N(11)#1	128.78(8)	O(2)#1-Tb(1)-N(2)	84.75(5)
O(4)#1-Tb(1)-N(11)#1	83.05(8)	O(4)-Tb(1)-N(1)#1	85.78(8)
O(1)#1-Tb(1)-N(11)#1	122.74(8)	O(4)#1-Tb(1)-N(1)#1	65.65(8)
O(1)-Tb(1)-N(11)#1	70.00(8)	O(1)#1-Tb(1)-N(1)#1	25.62(7)
N(12)#1-Tb(1)-N(11)#1	64.20(8)	O(1)-Tb(1)-N(1)#1	135.27(7)
N(12)-Tb(1)-N(11)#1	89.16(8)	N(12)#1-Tb(1)-N(1)#1	139.68(8)
N(11)-Tb(1)-N(11)#1	147.39(11)	N(12)-Tb(1)-N(1)#1	70.75(8)
O(4)-Tb(1)-O(2)	66.30(8)	N(11)-Tb(1)-N(1)#1	91.58(7)
O(4)#1-Tb(1)-O(2)	103.72(8)	N(11)#1-Tb(1)-N(1)#1	97.13(7)
O(1)#1-Tb(1)-O(2)	124.45(7)	O(2)-Tb(1)-N(1)#1	148.66(7)
O(1)-Tb(1)-O(2)	50.91(7)	O(2)#1-Tb(1)-N(1)#1	25.37(7)
N(12)#1-Tb(1)-O(2)	68.37(8)	N(2)-Tb(1)-N(1)#1	74.34(5)

Table S1: CHN Elemental Analysis of $[Tb(bipy)_2(NO_3)_3]$ complex.

	C [%]	H [%]	N [%]
Calculated	36.6	2.5	14.9
Found	36.2	2.5	15.0

Table S5: Bond lengths [Å] and angles [°].

N(1)-O(3)	1.220(3)	N(1)-O(1)	1.282(3)
N(1)-O(2)	1.262(3)	O(2)-N(1)-O(1)	116.2(2)
O(3)-N(1)-O(1)	121.2(3)	O(3)-N(1)-O(2)	122.6(3)
N(2)-O(5)	1.200(7)	N(2)-O(4)	1.279(3)
N(2)-O(4)#1	1.279(3)	O(5)-N(2)-O(4)	122.66(19)
O(5)-N(2)-O(4)#1	122.66(19)	O(4)#1-N(2)-O(4)	114.7(4)
N(11)-C(11)	1.339(4)	C(14)-C(15)	1.388(5)
N(11)-C(15)	1.357(4)	C(15)-C(16)	1.485(5)
N(12)-C(20)	1.343(4)	C(16)-C(17)	1.383(4)
N(12)-C(16)	1.351(4)	C(17)-C(18)	1.385(5)
C(11)-C(12)	1.391(5)	C(18)-C(19)	1.373(5)
C(12)-C(13)	1.371(5)	C(19)-C(20)	1.380(4)
C(13)-C(14)	1.382(5)		
C(11)-N(11)-C(15)	117.8(3)	C(14)-C(15)-C(16)	122.4(3)
C(20)-N(12)-C(16)	118.4(3)	N(12)-C(16)-C(17)	121.3(3)
N(11)-C(11)-C(12)	123.2(3)	N(12)-C(16)-C(15)	116.1(3)
C(13)-C(12)-C(11)	118.5(3)	C(17)-C(16)-C(15)	122.6(3)
C(12)-C(13)-C(14)	119.4(3)	C(16)-C(17)-C(18)	119.7(3)
C(13)-C(14)-C(15)	119.3(3)	C(19)-C(18)-C(17)	119.0(3)
N(11)-C(15)-C(14)	121.8(3)	C(18)-C(19)-C(20)	118.7(3)
N(11)-C(15)-C(16)	115.9(3)	N(12)-C(20)-C(19)	123.0(3)

Symmetry transformations used to generate equivalent atoms: A: -x+1,y,-z+1/2

Table S6: Anisotropic displacement parameters ($Å^2$x 10^3). The anisotropic displacement factor exponent takes the form: $-2\pi^2[h^2 a^{*2}U^{11} + ... + 2 h k a^* b^* U^{12}]$

	U^{11}	U^{22}	U^{33}	U^{23}	U^{13}	U^{12}
Tb(1)	25(1)	31(1)	34(1)	0	1(1)	0
N(1)	35(1)	46(1)	38(1)	-4(1)	-2(1)	-6(1)
O(1)	33(1)	46(1)	37(1)	0(1)	3(1)	-4(1)
O(2)	30(1)	44(1)	40(1)	-3(1)	4(1)	-7(1)
O(3)	54(2)	98(2)	42(1)	-10(1)	-7(1)	-27(1)
N(2)	54(4)	38(3)	53(3)	0	-20(2)	0
O(4)	48(2)	42(1)	52(1)	-10(1)	-4(1)	8(1)
O(5)	148(6)	32(2)	85(4)	0	-46(3)	0
N(11)	30(1)	35(1)	36(1)	-1(1)	3(1)	-2(1)
N(12)	33(1)	33(1)	34(1)	0(1)	1(1)	0(1)
C(11)	38(2)	41(2)	38(2)	-4(1)	3(2)	-3(1)
C(12)	36(2)	48(2)	49(2)	0(1)	12(2)	2(1)
C(13)	32(2)	48(2)	57(2)	6(2)	9(1)	-2(1)
C(14)	31(2)	46(2)	48(2)	4(1)	1(2)	-6(1)
C(15)	32(2)	35(1)	38(2)	5(1)	0(2)	-4(1)
C(16)	34(2)	36(2)	37(2)	6(1)	-2(1)	-2(1)
C(17)	42(2)	49(2)	48(2)	-3(2)	-1(2)	-12(1)
C(18)	58(2)	45(2)	46(2)	-7(2)	-4(2)	-11(2)
C(19)	56(2)	39(2)	39(2)	-6(1)	1(2)	2(2)
C(20)	39(2)	37(2)	42(2)	-1(1)	3(1)	0(1)

Table S7: Hydrogen coordinates (x 10^4) and isotropic displacement parameters ($Å^2$x 10^3).

	x	y	z	U(eq)
H(11)	3789	6543	4270	47
H(12)	2444	6500	4680	53
H(13)	1553	5046	3858	54
H(14)	2041	3588	2687	50
H(17)	2610	2000	1834	55
H(18)	3245	603	721	60
H(19)	4550	1227	296	54
H(20)	5193	3163	1016	47

Table S8: Hydrogen bonds [Å and °].

D-H...A	d(D-H)	d(H...A)	d(D...A)	<(DHA)
C(11)-H(11)...O(2)	0.95	2.37	2.976(4)	121.5
C(12)-H(12)...O(3)#2	0.95	2.45	3.239(4)	140.3
C(14)-H(14)...O(1)#3	0.95	2.60	3.552(4)	174.9
C(17)-H(17)...O(1)#3	0.95	2.43	3.319(4)	156.1
C(20)-H(20)...O(3)#4	0.95	2.58	3.372(4)	141.4

Symmetry transformations used to generate equivalent atoms:
#1 -x+1,y,-z+1/2; #2 x-1/2,-y+3/2,-z+1; #3 x-1/2,y-1/2,-z+1/2; #4 x,-y+1,z-1/2

Table S2: Bond distances from the metal center to the ligand in the compounds [Tb(bipy)$_2$(NO$_3$)$_3$] compared to the literature values of the compound [Eu(bipy)$_2$(NO$_3$)$_3$].[10]

Eu-N	2.554(5)	Tb-N	2.5240(24)
	2.540(5)		2.5209(26)
Eu-O	2.494(4)	Tb-O	2.4719(21)
	2.561(5)		2.5510(25)
	2.491(5)		2.4685(27)

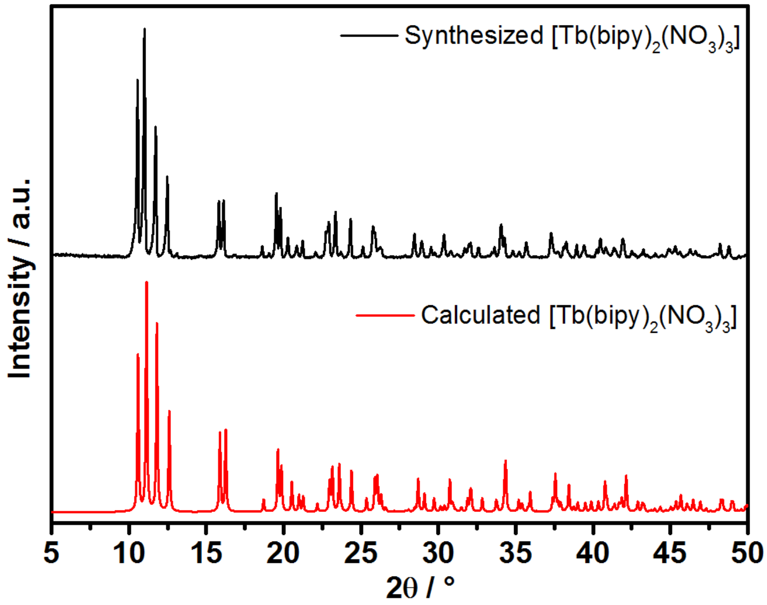

Figure S4: Comparison between X-ray diffraction patterns of synthesized (Experiment **1**, black curve) and calculated (red curve) [Tb(bipy)$_2$(NO$_3$)$_3$], based on single crystal diffraction measurements.

3. *In situ* monitoring the crystallization process

3.1. Solution addition at 0.5 mL/min

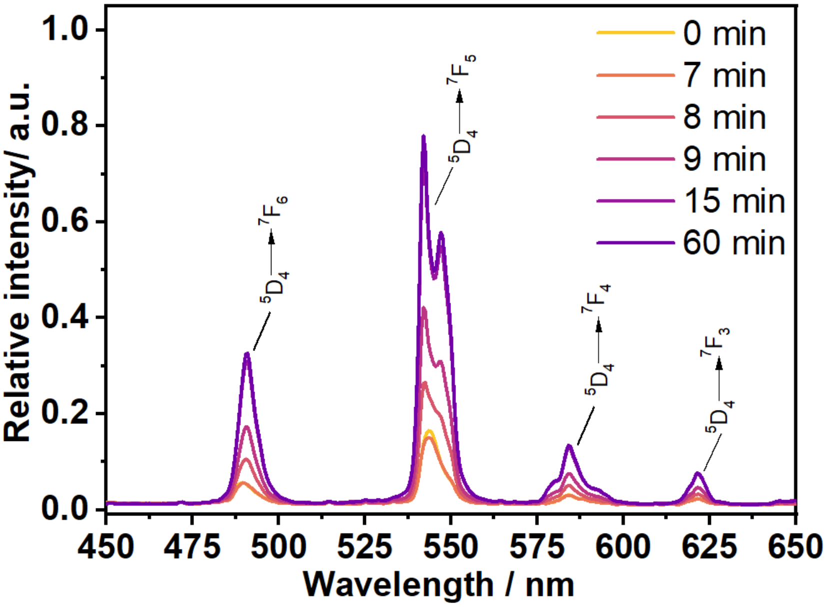

Figure S5: Time-dependent emission intensity (λ_{ex} = 365 nm) recorded during the synthesis of [Tb(bipy)$_2$(NO$_3$)$_3$] with the addition rate of 2,2'-bipyridine at 0.5 mL/min (Exp. **2**, Table 1).

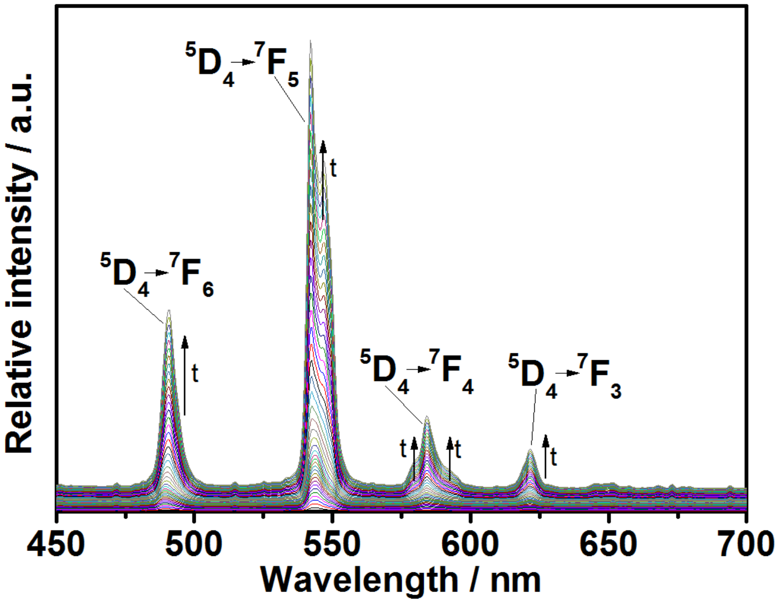

Figure S6: Peak-splitting of the time-dependent *in situ* emission spectra (λ_{ex} = 365 nm) recorded during the synthesis of [Tb(bipy)$_2$(NO$_3$)$_3$] with the addition rate of 2,2'-bipyridine at 0.5 mL/min (Experiment **2**, Table 1).

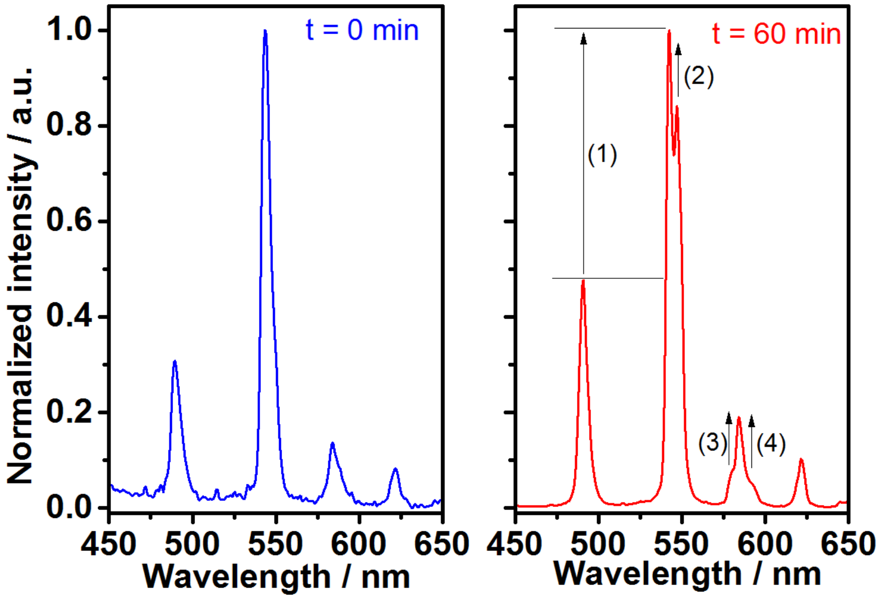

Figure S7: *In situ* emission intensity (λ_{ex} = 365 nm) recorded during the synthesis of [Tb(bipy)$_2$(NO$_3$)$_3$] with the addition rate of 2,2'-bipyridine at 0.5 mL/min (Experiment **2**, Table 1).

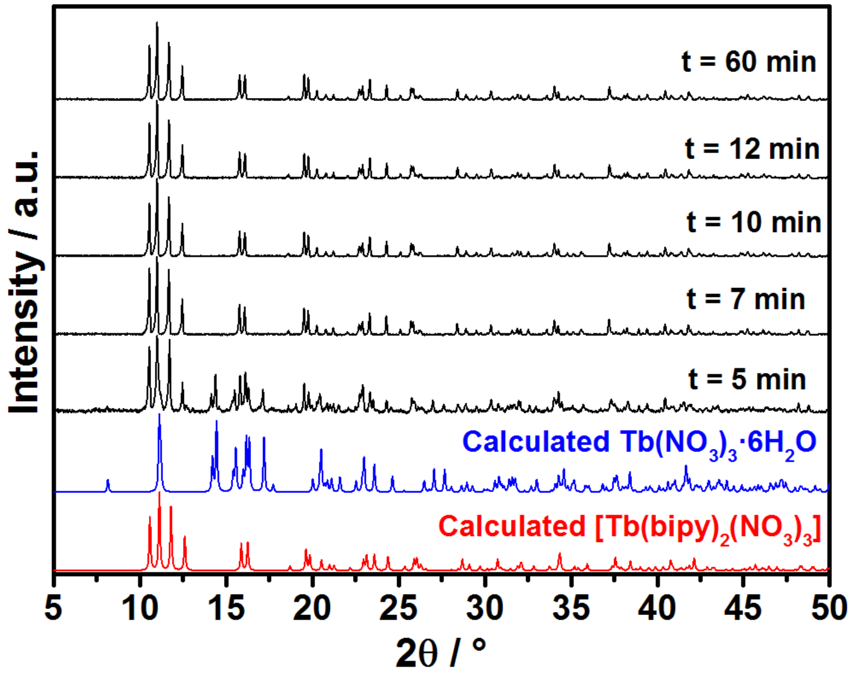

Figure S8: *Ex situ* XRD analysis of samples removed from the reactor during the synthesis of [Tb(bipy)$_2$(NO$_3$)$_3$] with the addition rate of 2,2'-bipyridine at 0.5 mL/min (Experiment **2**, Table S1).

3.2. Solution addition at 10 mL/min

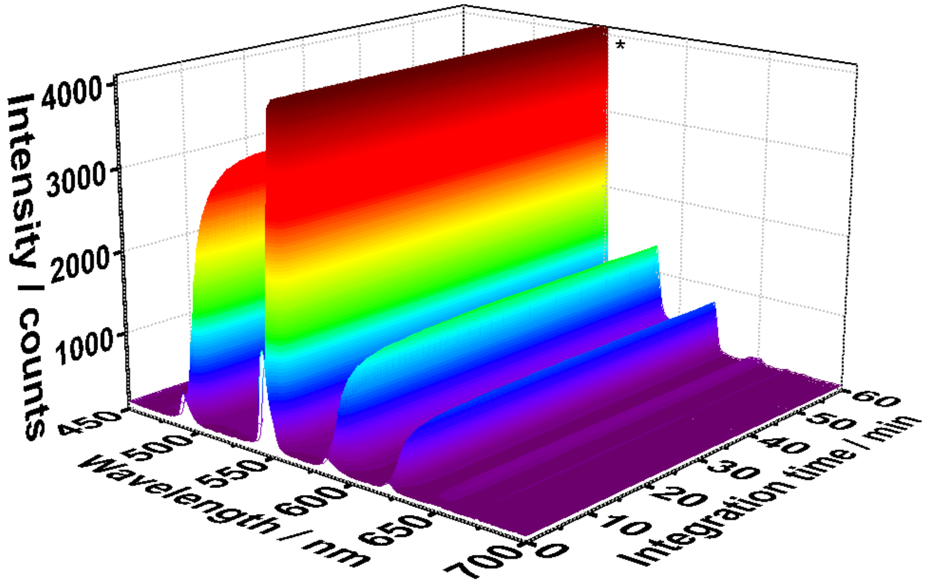

Figure S9: *In situ* emission intensity (λ_{ex} = 365 nm) recorded during the synthesis of $[Tb(bipy)_2(NO_3)_3]$ with the addition rate of 2,2'-bipyridine at 10 mL/min (Experiment **3**, Table 1).

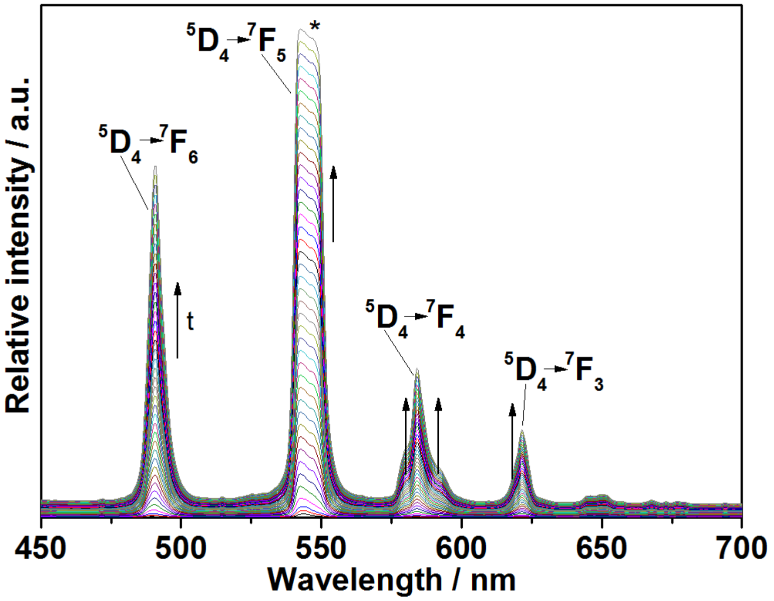

Figure S10: *In situ* emission intensity (λ_{ex} = 365 nm) recorded during the synthesis of $[Tb(bipy)_2(NO_3)_3]$ with the addition rate of 2,2'-bipyridine at 10 mL/min (Experiment **3**, Table 1).

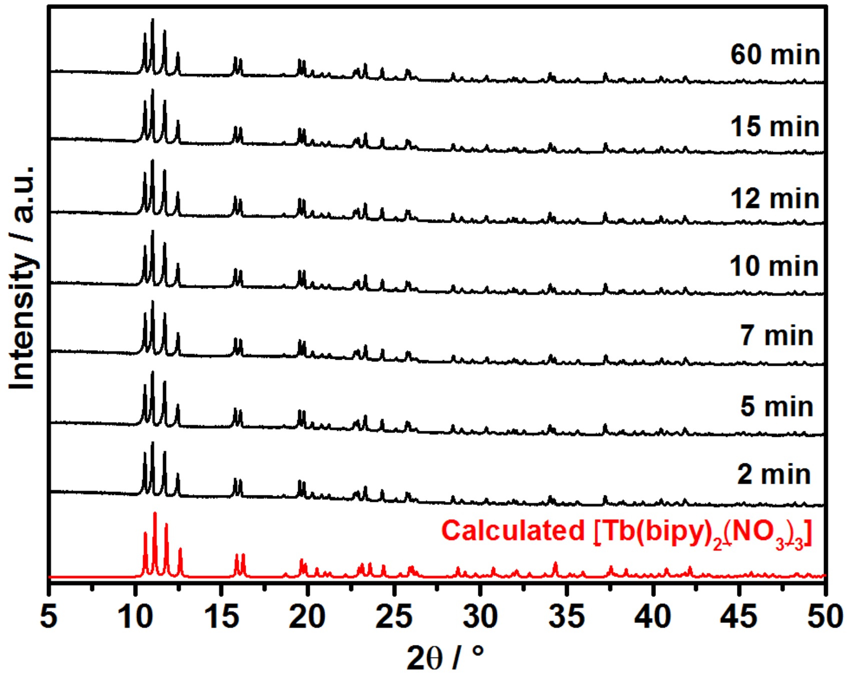

Figure S11: X-ray diffraction patterns of samples removed at t = 2, 5, 7, 10, 15, 15 and 60 min from the reactor in comparison to the calculated pattern for $[Tb(bipy)_2(NO_3)_3]$ (Experiment 3, Table 1).

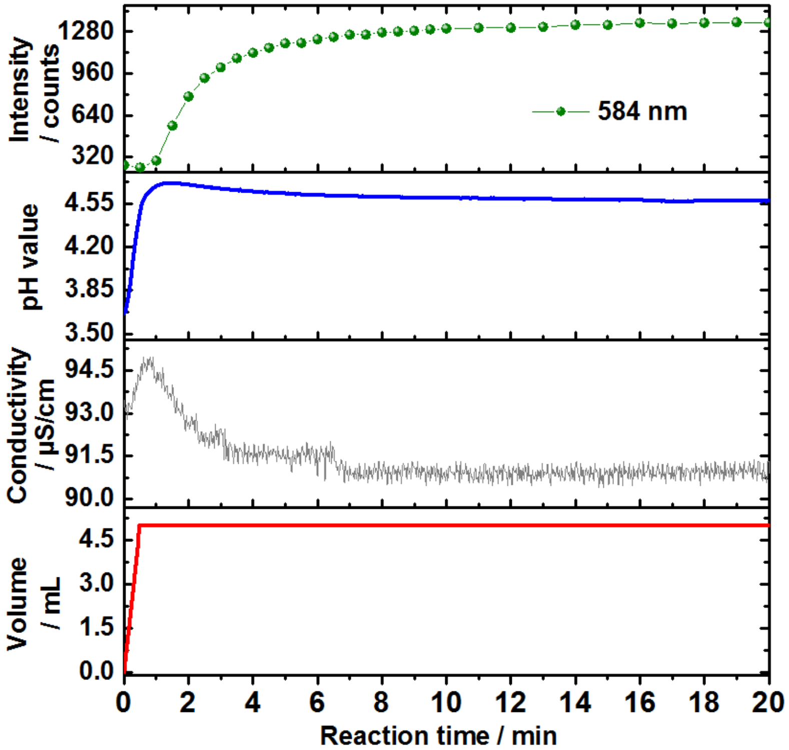

Figure S1: Time-dependent emission intensity of the Tb^{3+} $^5D_4 \rightarrow {}^7F_4$ transition at 584 nm (green dotted curve) in comparison to simultaneous *in situ* measurements of pH value (blue curve), ionic conductivity (gray curve) as well as the addition rate of the bipy solution to the reactor containing terbium(III) nitrate at 10 mL/min (red curve, Experiment **3**, Table 1).

4. Simultaneous *in situ* luminescence and powder X-ray diffraction methods

4.1. Bipy:Tb^{3+} ratio 1.5:1

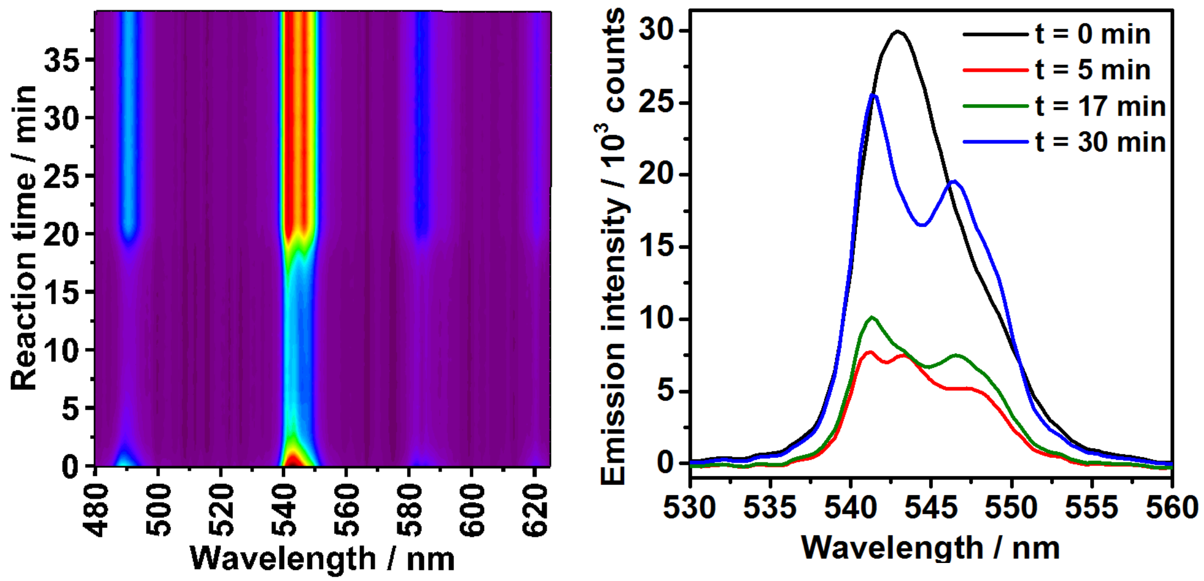

Figure S2: *In situ* luminescence measurements recorded simultaneous to *in situ* XRD at the P08 DESY beamline during the synthesis of $[Tb(bipy)_2(NO_3)_3]$ (ratio bipy:Tb = 1.5:1, Experiment **5**, Table 1).

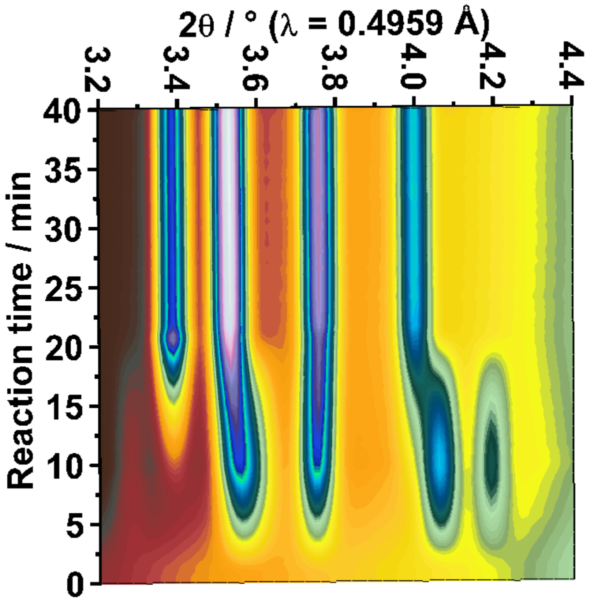

Figure S3: *In situ* XRD measurements recorded at the P08 DESY beamline (λ = 0.4959 Å) during the synthesis of $[Tb(bipy)_2(NO_3)_3]$ (ratio bipy:Tb = 1.5:1, Experiment **5**, Table S1).

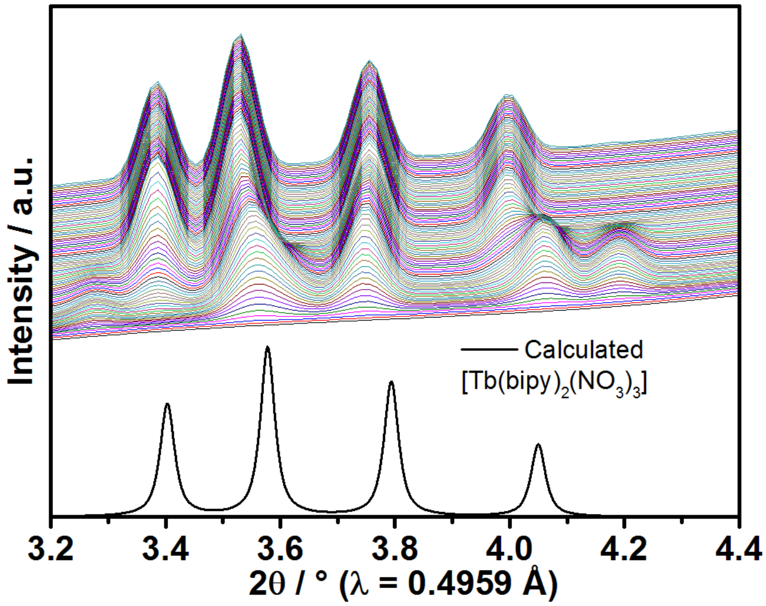

Figure S4: Measured *in situ* XRD patters in comparison to calculated diffraction patterns for $[Tb(bipy)_2(NO_3)_3]$ (ratio bipy:Tb = 1.5:1, Experiment **5**, Table S1).

4.2. Bipy:Tb^{3+} ratio 1:1

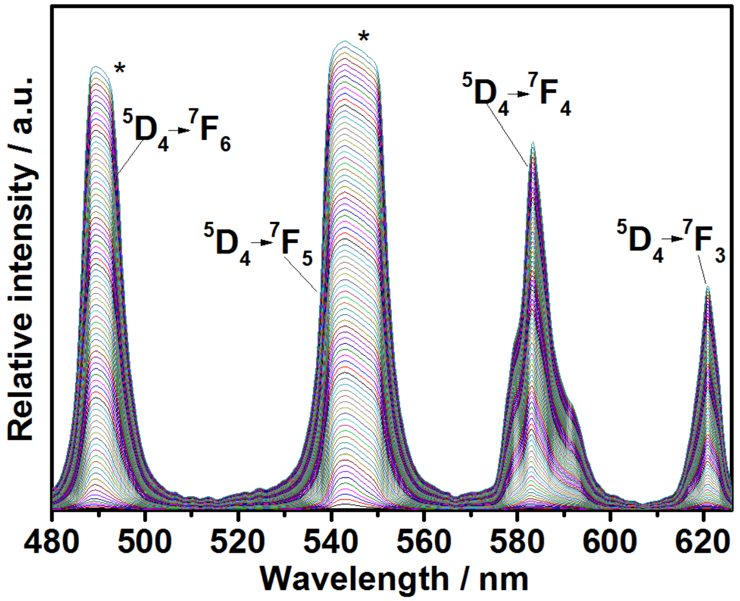

Figure S5: *In situ* emission intensity (λ_{ex} = 365 nm) recorded during the synthesis of $[Tb(bipy)_2(NO_3)_3]$ with the addition rate of 2,2'-bipyridine at 0.5 mL/min (ratio bipy:Tb = 1:1, Experiment **6**, Table **1**).

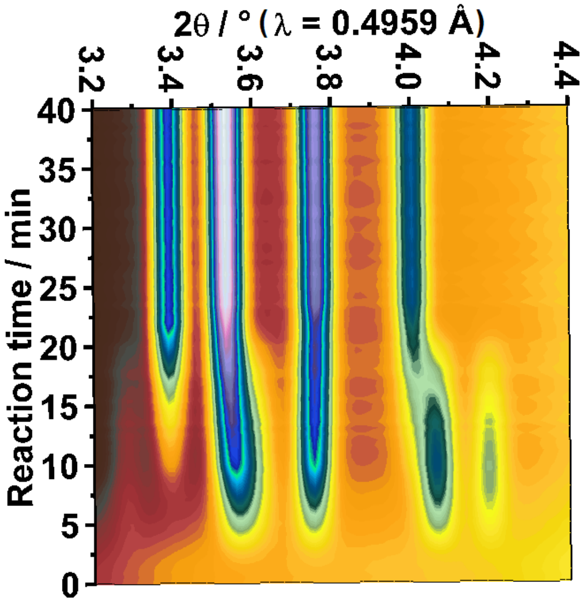

Figure S6: *In situ* XRD measurements recorded at the P08 DESY beamline (λ = 0.4959 Å) during the synthesis of [Tb(bipy)$_2$(NO$_3$)$_3$] (ratio bipy:Tb = 1:1, Experiment **6**, Table **1**).

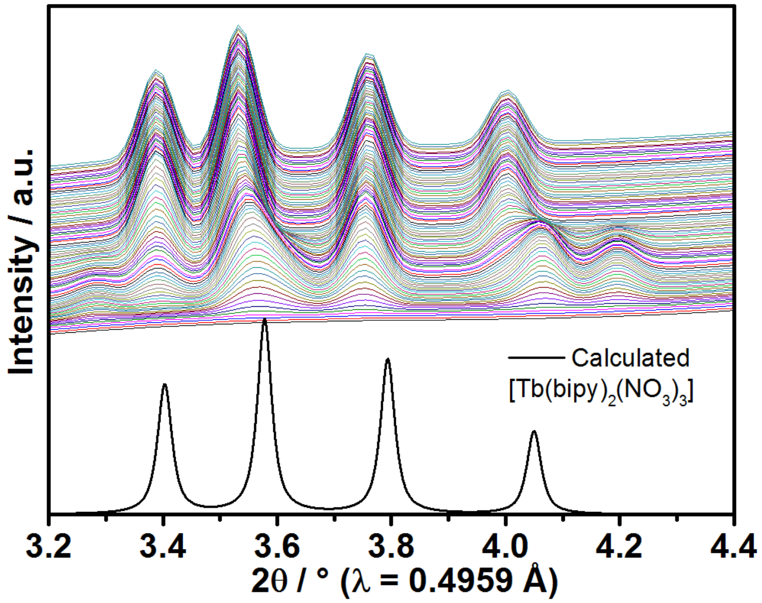

Figure S7: Measured *in situ* XRD patters in comparison to calculated diffraction patterns for [Tb(bipy)$_2$(NO$_3$)$_3$] (ratio bipy:Tb = 1:1, Experiment **6**, Table **1**).
